# Diagnostics and analysis of SARS-CoV-2: current status, recent advances, challenges and perspectives

**DOI:** 10.1039/d2sc06665c

**Published:** 2023-05-03

**Authors:** Tao Dong, Mingyang Wang, Junchong Liu, Pengxin Ma, Shuang Pang, Wanjian Liu, Aihua Liu

**Affiliations:** a Institute for Chemical Biology & Biosensing, College of Life Sciences, Qingdao University 308 Ningxia Road Qingdao 266071 China liuah@qdu.edu.cn; b School of Pharmacy, Medical College, Qingdao University 308 Ningxia Road Qingdao 266071 China; c Qingdao Hightop Biotech Co., Ltd 369 Hedong Road, Hi-tech Industrial Development Zone Qingdao 266112 China

## Abstract

The disastrous spread of severe acute respiratory syndrome coronavirus 2 (SARS-CoV-2) has induced severe public healthcare issues and weakened the global economy significantly. Although SARS-CoV-2 infection is not as fatal as the initial outbreak, many infected victims suffer from long COVID. Therefore, rapid and large-scale testing is critical in managing patients and alleviating its transmission. Herein, we review the recent advances in techniques to detect SARS-CoV-2. The sensing principles are detailed together with their application domains and analytical performances. In addition, the advantages and limits of each method are discussed and analyzed. Besides molecular diagnostics and antigen and antibody tests, we also review neutralizing antibodies and emerging SARS-CoV-2 variants. Further, the characteristics of the mutational locations in the different variants with epidemiological features are summarized. Finally, the challenges and possible strategies are prospected to develop new assays to meet different diagnostic needs. Thus, this comprehensive and systematic review of SARS-CoV-2 detection technologies may provide insightful guidance and direction for developing tools for the diagnosis and analysis of SARS-CoV-2 to support public healthcare and effective long-term pandemic management and control.

## Abstract 2

This review presents the recent advances in SARS-CoV-2 detection, including molecular diagnostics, antigen and antibody testing, and advances assay of neutralizing antibodies and emerging variants.

## Graphical abstract

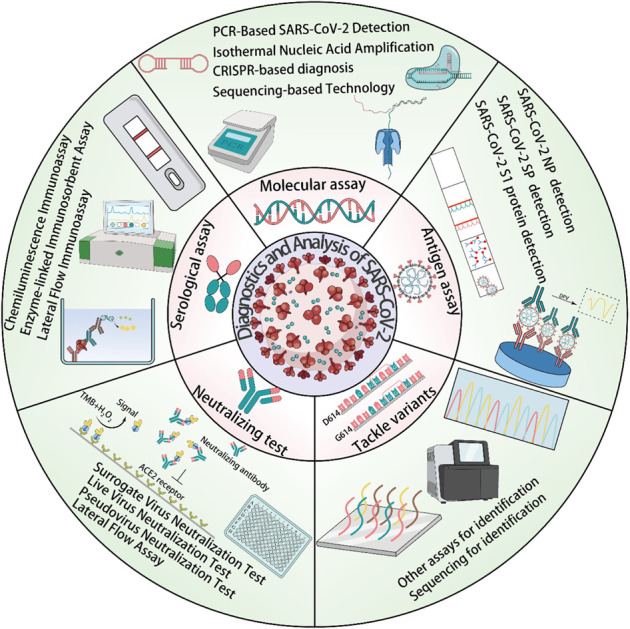

## 1. Introduction

Since the outbreak of coronavirus disease 2019 (COVID-19) caused by severe acute respiratory syndrome coronavirus 2 (SARS-CoV-2) in December 2019, this virus has spread rapidly around the world.^1,2^ Consequently, the rampant COVID-19 has induced severe public health problems, threatening the lives and security of people around the world and putting stress on national healthcare systems. As of March 2023, over 6.8 million people died of COVID-19 globally, according to a report by the World Health Organization (WHO). In addition, SARS-CoV-2 shows the tendency of long-term coexistence with humans and continues to mutate, generating new variants and subvariants, which induce enhanced immune escape and rapid transmission, according to the reports by the US Centers for Disease Control and Prevention (CDC) (https://www.cdc.gov). The shutdown and delayed resumption of production caused by the epidemic have seriously weakened the global economy and led to various social “after-effects” (reduced social workforce and enlarged wealth gap).

**Fig. 1:**
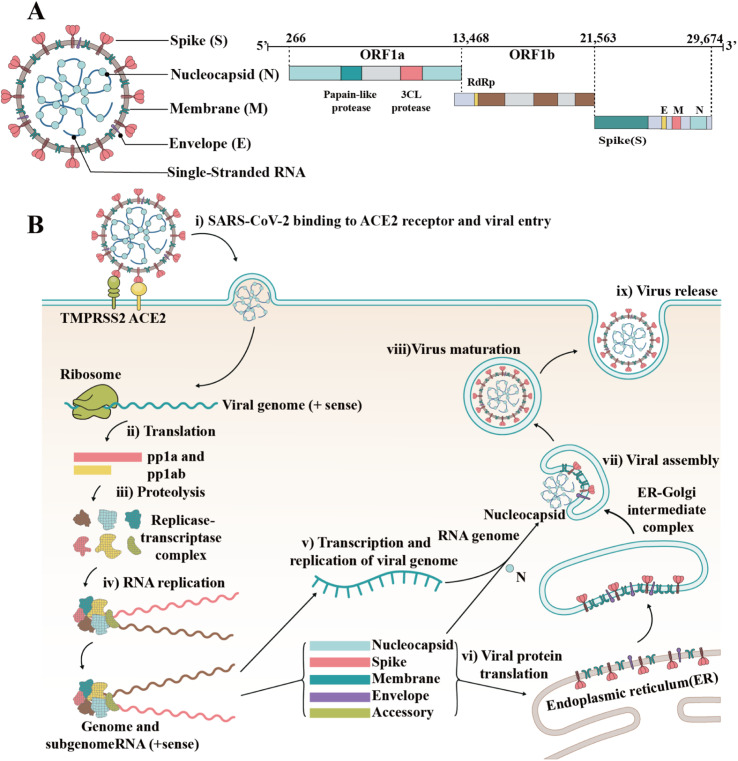
Structure and invasion mechanism of SARS-CoV-2. (A) Genomic structure of coronaviruses is highly conserved and includes three main regions. ORFs 1a and 1b contain two polyproteins that encode the nonstructural proteins (nsp), which include enzymes such as RdRp. The last third of the genome encodes structural proteins. Accessory genes can also be interspersed throughout the genome. (B) After the S protein binds to ACE2, the viral genome is fused with the plasma membrane *via* TMPRSS2 cleavage and activation. Once inside the host cell, the virus may interact with cellular enzymes such as non-structural protein 11 (PP1a) and NSP16 (PP1ab), which can remove the phosphate groups from proteins, interact with SARS-CoV-2 during infection, and potentially regulate the virus replication cycle by affecting the phosphorylation and gene expression of viral proteins. Subsequently, the virus uses its RNA genome to hijack the machinery of the host cell to replicate rapidly and produce new virus particles. The newly formed virus particles assemble and are released from the infected cell, often destroying the host cell in the process.

SARS-CoV-2 is an enveloped single-stranded RNA virus.^3,4^ Its genome length is 29 881 nucleotides (nt) (GenBank number MN908947). The gene fragments express the structural and nonstructural proteins, where the spike (S), envelope (E), membrane (M) and nucleocapsid (N) genes encode the structural proteins of the S protein (SP), E protein (EP), M protein (MP), and N protein (NP), respectively, while the open reading frame (ORF) region encodes nonstructural proteins, such as 3-chymotrypsin-like protease, papain-like protease and RNA-dependent RNA polymerase (RdRP) (Fig. 1A).^5,6^ As a type I fusion protein, SP plays a crucial role in the process of virus infection and pathogenesis.^7^ SP consists of subunit S1 and subunit S2, of which subunit S1 is composed of the N-terminal domain (NTD) and receptor-binding domain (RBD), while subunit S2 mediates the fusion between the virus and the cell membrane. The RBD of the S protein can bind to the cell receptor of angiotensin-converting enzyme 2 (ACE2), which is the key to the viral invasion of the body.^8,9^ When S proteins bind to the receptor, transmembrane serine protease 2 (TMPRSS2), located on the host cell membrane, facilitates virus entry in the cell by activating S proteins.^10^ Once the virus enters the cell, it releases its RNA, which begins to replicate in the host cell. Replication and transcription of the viral RNA genome occur through protein cleavage and assembly of the replicase–transcriptase complex. After replication of viral RNA, the synthesis of structural proteins, assembly and packaging in the host cell, the viral particles are released (Fig. 1B).^11,12^

Since SARS-CoV-2 became popular at the end of 2019, many different subtypes or branches have evolved and spread globally. The mutation rate of this virus is about 2 nt per month, which is much lower than the influenza (4 nt per month) or human immunodeficiency virus (HIV) (8 nt per month).^13^ Accordingly, with the discovery of new variants of SARS-CoV-2, it is necessary to periodically reconfigure diagnostic tests for this virus.^14^ Among the SARS-CoV-2 mutant strains that have been detected and isolated, the main types of mutations were found to occur in RBD of the S protein.^15,16^ Mutations in this structure may increase the affinity with the receptor,^17,18^ weaken the effect of neutralizing antibodies,^19,20^ or cause the virus to escape immunity.^21,22^ Although mutations are also present in the N protein, compared to the S protein, it is highly conserved and often used as a rapid detection marker for SARS-CoV-2. Since December 2020, four rapidly spreading virus lineages have been identified as variants of concern (VOCs), marking the entry of the pandemic into a new phase. The four VOCs are the Alpha, Beta, Gamma and Delta variants, while many of the other variants that have subsequently emerged possess some of the mutational characteristics of these four variants. Currently, the B.1.1.529 Pango lineage (Omicron variant), including BA.1, BA.2, BA.3, BA.4, BA.5, and its descendent lineages, are prevalent. The WHO has emphasized that these descendant lineages should be monitored, and their virus characteristics should be examined.

The incubation period of COVID-19 ranges from 1 to 14 days, mostly 3 to 7 days with the main symptoms of fever, dry cough and fatigue.^23–25^ Some patients have diminished or lost sense of smell and taste as the first symptoms. A few patients have nasal congestion, runny nose, sore throat, conjunctivitis, myalgia and diarrhea.^26–28^ Severe patients usually develop respiratory distress and/or hypoxemia one week after the onset of the disease. Furthermore, they can rapidly progress to acute respiratory distress syndrome, septic shock, difficult-to-correct metabolic acidosis, coagulation dysfunction, organ failure, *etc.*^29^ Notably, many infected individuals may not have significant clinical symptoms due to the decreased virulence of this virus.^30,31^ Although SARS-CoV-2 infection will not be as fatal as its initial outbreak, studies have shown that many infected people suffered from severe sequelae.^32–34^ According to the CDC, over 80% of the nearly 24 million adults in the United States with long COVID experience difficulties in daily activities.^2^ Moreover, a report by the Brookings Institution also indicates that approximately 4 million people in the United States are unemployed because of long COVID symptoms.^35^

The spread of SARS-CoV-2 and the shocking infection data caused the world to seek effective prevention, control, and treatment methods. Thus, to help researchers in different fields have a more comprehensive understanding of the SARS-CoV-2 assays and promote the development of new assay technologies, herein, we review the articles related to SARS-CoV-2 detection technologies from 2020 to March 2023. Compared with the previous reviews, we not only elaborate on the principles of each method but also summarize its utilization, advantages and limits. In response to the simplicity and broadness of the published reviews on SARS-CoV-2 antigen detection, we overview this type of technique in terms of various antigenic subunits and analyze its applications in clinical settings. This makes our article more systematic, insightful and practically meaningful. In addition to the in-depth insight into the three main types of assays (molecular assays, serological assays, and antigen assays), we also review the corresponding countermeasure strategies for SARS-CoV-2 variants. Further, the mutational location characteristics and the epidemiological features of various variants are summarized. Meanwhile, related detection and countermeasure strategies for different mutation locations are also proposed, which are of great significance for diagnosing and preventing mutant strains. Finally, the challenges and possible strategies are prospected to explore new assays to meet different diagnostic needs.

## 2. Molecular assays for the detection of viral nucleic acids

SARS-CoV-2 molecular assays are the most direct and sensitive detection methods. Currently, more than 500 commercial kits are available worldwide for the molecular detection of SARS-CoV-2 RNA, and the emergence of these kits has played a critical role in the containment of the SARS-CoV-2 outbreak (Table 1).

**Table 1:** Examples of commercial molecular diagnostic assays for the detection of SARS-CoV-2^a^

Manufacturer	Test name	Method	Target gene	Specimen type	LOD^b^	Sensitivity	Specificity	Status^c^	Ref.
LumiraDx UK Ltd	LumiraDx SARS-CoV-2 RNA STAR Complete	RT, qSTAR amplification, home collection, screening, pooling	N gene	Nasal swabs	32 TCID_50_ per mL	97%	96%	H	36
Abbott Molecular Inc.	Abbott RealTime SARS-CoV-2 Assay	RT-PCR	N and RdRP genes	Nasopharyngeal and oropharyngeal swabs	100 copies per mL	95.9%	100%	H	37
Meridian Bioscience, Inc.	Revogene SARS-CoV-2	RT-PCR	N gene	Nasopharyngeal, oropharyngeal, anterior nasal, and mid-turbinate nasal swab specimens	7 TCID_50_ per mL	97.7%	97.7%	H, M	15
Talis Biomedical Corporation	Talis One COVID-19 Test System	Reverse transcriptase isothermal amplification	ORF1ab gene, N gene	Nasal mid-turbinate swabs	500 copies per mL	100%	100%	H, M, W	38
Cepheid	Xpert Xpress CoV-2/Flu/RSV plus	rRT-PCR	N gene, E gene, RdRP genes	Nasopharyngeal swabs, anterior nasal swabs, nasal wash/aspirate specimens	138 copies per mL	100%	100%	H, M, W	39
Life Technologies Corporation (a part of Thermo Fisher Scientific Inc.)	TaqPath COVID-19 Fast PCR Combo Kit 2.0	rRT-PCR	N gene, ORF1a, ORF1b	Saliva	1000 GCE per mL	97.1%	97.6%	H	40
Detect, Inc.	Detect Covid-19 Test	RT-LAMP	ORF1ab	Anterior nasal swabs	800 copies per mL	90.9%	97.5%	Home, H, M, W	41
PerkinElmer, Inc.	PKamp Respiratory SARS-CoV-2 RT-PCR Panel 1	rRT-PCR	N gene, ORF1a gene	Nasopharyngeal swabs, anterior nasal swabs, and mid-turbinate swabs	SARS-CoV-2: 0.11 TCID_50_ per mL	100%	100%	H	20
LMSI, LLC (dba Lighthouse Lab Services)	CovidNow SARS-CoV-2 Assay	rRT-PCR	N gene	Upper respiratory specimens, self-collected anterior nasal swab specimens	800 copies per mL	96%	100%	H	21
Applied DNA Sciences Inc.	Linea COVID-19 Assay Kit	rRT-PCR	S gene (S1 and S2 regions)	Nasopharyngeal swabs and oropharyngeal swabs	1250 copies per mL	98%	92%	H	22
Enzo Life Sciences, Inc.	AMPIPROBE SARS-CoV-2 Test System	rRT-PCR	N gene and RdRP gene	Nasal, mid-turbinate, nasopharyngeal, and oropharyngeal swab specimens	280 copies per mL	96.2%	98%	H	42
Thermo Fisher Scientific Inc.	TaqPath COVID-19 RNase P Combo Kit 2.0	rRT-PCR	N gene, ORF1a, ORF1b	Nasopharyngeal, anterior, and mid-turbinate nasal swabs	75 GCE per mL	96.7%	95%	H	43
Twist Bioscience Corporation	SARS-CoV-2 NGS Assay	NGS	Several	Nasopharyngeal swabs, oropharyngeal swabs, anterior nasal swabs	800 copies per mL	96.7%	100%	H	44
BioGX, Inc.	BioGX Xfree COVID-19 Direct RT-PCR	rRT-PCR	N1 gene	Nasopharyngeal, anterior nasal, mid-turbinate	1000–3000 copies per mL (depending on the platform used)	95%	100%	H	26
OSANG Healthcare	GeneFinder COVID-19 Plus RealAmp Kit	rRT-PCR	RdRP, E gene, N gene	Alveolar lavage fluid, throat swab, sputum	10 copies per 20 μL reaction	100%	100%	H	45
OPTI Medical Systems	OPTI SARS-CoV-2 RT PCR Test	rRT-PCR, pooling	N gene (N1 and N2)	Anterior nasal swabs, mid-turbinate nasal swabs, nasopharyngeal swabs	700–900 copies per mL	100%	100%	H	46
PathogenDx, Inc.	DetectX-Rv	rRT-PCR, DNA microarray hybridization	N gene (N1 and N2 regions)	Nasopharyngeal swabs, oropharyngeal swabs, mid-turbinate swabs, anterior nasal swabs	1000 copies per mL	95%	100%	H	47
Hologic, Inc.	Aptima SARS-CoV-2 Assay	Real-time TMA, dual kinetic assay	ORF1ab (2 targets)	Nasopharyngeal, nasal, mid-turbinate, and oropharyngeal swab specimens	212 copies per mL within a sample (83 copies per mL within the tested aliquot)	100%	98%	H	48
LumiraDx UK Ltd	LumiraDx SARS-CoV-2 RNA STAR Complete	RT-qSTAR amplification	ORF1a	Mid-turbinate, nasopharyngeal, and oropharyngeal swabs	7500 copies per mL	97.8%	98.5%	H	49
Quidel Corporation	Solana SARS-CoV-2 Assay	Molecular isothermal Reverse Transcriptase – Helicase-Dependent Amplification (RT-HDA)	ORF1ab	Nasopharyngeal and nasal swab specimens	1.16 ×10^4^ copies per mL	90.3%	97.8%	H, M	32
Cue Health Inc.	Cue COVID-19 Test for Home and Over the Counter Use	Isothermal amplification, home collection	N gene	Anterior nares swabs	2700 copies per mL	96% (symptomatic), (100% asymptomatic)	98% (symptomatic), (100% asymptomatic)	Home, H, M, W	50
Grifols Diagnostic Solutions Inc.	Procleix SARS-CoV-2 Assay	Transcription-mediated nucleic acid amplification (TMA)	Not stated	Anterior nasal and mid-turbinate nasal swabs, nasopharyngeal	60 copies per mL	100%	100%	H	51
Agena Bioscience, Inc.	MassARRAY SARS-CoV-2 Panel	rRT-PCR, MALDI-TOF	N gene (N1, N2, and N3 regions), ORF1, and ORF1ab	Nasopharyngeal swabs, oropharyngeal swabs, nasal and mid-turbinate swabs	310–2500 copies per mL	98.9%	100%	H	35
Cepheid	Xpert® Omni SARS-CoV-2	rRT-PCR	E gene and N gene (N2 region)	Nasopharyngeal, oropharyngeal, nasal, or mid-turbinate swab specimens	400 copies per mL	100%	100%	H, M, W	52
Lucira Health, Inc.	Lucira COVID-19 All-In-One Test Kit	RT-LAMP	N gene (two regions)	Nasal swab (self-collection), nasal swab (healthcare collection)	2700 copies per swab (or 900 copies per mL if the sample is 100% lysed and eluted)	94%	98%	Home, H, M, W	53
GenMark Diagnostics, Inc.	ePlex Respiratory Pathogen Panel 2	rRT-PCR, electrochemical detection	Not stated	Nasopharyngeal swabs	0.01TCID_50_ per mL	100%	100%	H, M	54
Seasun Biomaterials, Inc.	AQ-TOP COVID-19 Rapid Detection Kit PLUS	RT-LAMP	ORF1ab and N genes	Oropharyngeal and nasopharyngeal swabs, anterior nasal and mid-turbinate nasal swabs	1000 copies per mL	100%	100%	H	55
Clear Labs, Inc.	Clear Dx SARS-CoV-2 Test	rRT-PCR, NGS	Several	Nasopharyngeal swab, oropharyngeal swab, anterior nasal swab	2000 copies per mL	100%	100%	H	56
Abbott Diagnostics Scarborough	ID NOW COVID-19	Isothermal amplification	RdRP	Direct nasal secretions, nasopharyngeal or throat swabs	125 copies per mL	100%	100%	H, M, W	57
Cue Health Inc.	Cue COVID-19 Test	Isothermal amplification	N gene	Nasal swab	1300 copies per mL	100%	92%	Home, H, M, W	58

a H: laboratories certified under the Clinical Laboratory Improvement Amendments of 1988 (chemiluminescence immunoassay, CLIA), 42 U.S.C. §263a, that meet requirements to perform high complexity tests. M: laboratories certified under the Clinical Laboratory Improvement Amendments of 1988 (CLIA), 42 U.S.C. §263a, that meet requirements to perform moderate complexity tests. W: patient care settings operating under a CLIA Certificate of Waiver.

b LOD, Limit of detection.

c Status, Authorized settings include the following.

### 2.1 PCR-based SARS-CoV-2 detection

The foundation of molecular diagnosis depends on identifying and amplifying viral genetic material from suspected individual specimens. Reverse transcription-quantitative polymerase chain reaction (RT-qPCR) is the gold standard for identifying SARS-CoV-2, powered by its ability to amplify minuscule amounts of viral genetic information.^59,60^

#### 2.1.1 Reverse transcription-polymerase chain reaction (RT-PCR)

**Fig. 2:**
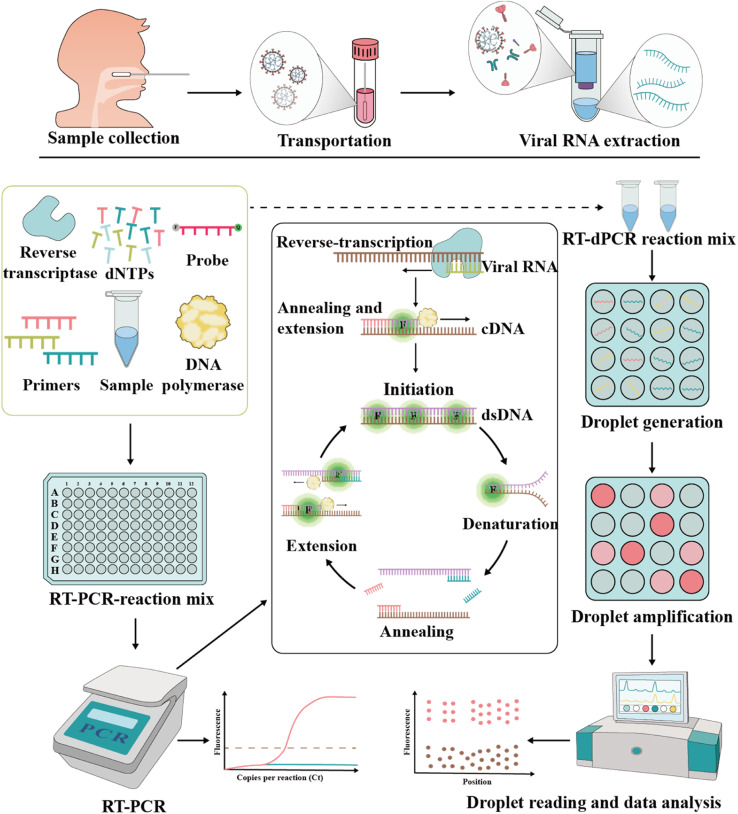
Schematic representation of RT-PCR and RT-dPCR procedures used to detect SARS-CoV-2. In both assays, appropriate specimens are collected, and viral RNA is extracted. In RT-PCR, the relative or absolute concentration of the target of interest is assessed by measuring the fluorescence signal, which shows the amplification in each cycle. In RT-dPCR, the absolute concentration of the target nucleic acid is determined based on the number of partitions that are either positive or negative for amplification based on fluorescence signals.

Currently, RT-PCR is the most frequently used molecular assay.^61,62^ In RT-PCR assays, RNA is first extracted from samples, and the rapid, high-throughput silica or magnetic particle method is preferred.^63–65^ After the extraction is complete, the SARS-CoV-2 RNA also needs to be reverse transcribed into complementary DNA (cDNA) strands, which are then mixed with primers, probes and reaction reagents on an automated thermal cycling system for amplification. Ultimately, the produced signal is commonly detected by fluorescence or electrical method (Fig. 2).^66,67^ Nowadays, both analysis kits are commercially available. In a one-step assay, reverse transcription and PCR amplification are integrated into a single reaction.^68,69^ In a two-step analysis, reactions are performed sequentially in separate tubes, which are more labor-intensive but more sensitive.^70,71^

The RT-PCR assay can target different genes^72^ such as RdRp gene, N gene, E gene, S gene and ORF1b or ORF8 region of the SARS-CoV-2 genome.^73^ Vogels *et al.* compared seven sets of primers provided by the WHO and found that they all had similar performances.^74^ Notably, Corman *et al.* indicated that different targets in the SARS-CoV-2 genome have different properties, affecting the sensitivity and specificity.^75^ Recently, many studies showed that primer probes for the detection of the N and E genes are more sensitive than that targeting the RdRp gene.^74,76–78^ This merit may be attributed to the mismatch of reverse primers used in detecting RdRp genes.^79^ To improve the diagnostic efficiency and reliability, two or more genes should be included in the RT-qPCR reaction to enhance the identification of true positives. In addition, mutations in target genes may affect the sensitivity of the assay, which may lead to false negative results.^80–82^ According to the sequence analysis of the SARS-CoV-2 genome by the Global Initiative of Sharing All Influenza Data (GISAID) database and China CDC, the N primer region has a higher rate of viral mutations compared to other primer sets.^83^ Although this does not imply that the primers cannot bind, it reveals the variability of the target region. A report showed that the deletion of S gene locations 69 and 70 in VOC B.1.1.7 failed at least one RT-PCR-based diagnostic kit for S gene detection.^61^ These findings highlight the importance of the independent evaluation of the primer-probe sets used in SARS-CoV-2 RT-PCR assays.

The RT-PCR assays of SARS-CoV-2 mainly use samples collected from the upper respiratory *via* swabs. Among the samples, sputum and nasal swabs have higher viral loads within seven days after symptom onset, whereas pharyngeal swabs are unreliable at eight days after symptom onset,^84–86^ given that viral loads are lower in samples after the 8th day. Generally, sputum samples have higher viral loads than pharyngeal swab samples, while urine or stool samples have lower viral RNA loads.^84,86–88^ After systematic evaluation of five studies, Woloshin *et al.* found that RT-PCR had a high rate of false negatives (2% to 29%).^89^ This phenomenon may be contributed from improper sampling techniques, low viral load in the sampling area, or mutations in the viral genome.^90–93^ Thus, to improve the sensitivity, safety and rapidity of RT-PCR assays, Erster *et al.* used a lysis buffer supplemented with nucleic acid stabilization and lysis buffer (NSLB) instead of the traditional viral transfer medium (VTM) for better sample preservation.^94^

Currently, the RT-PCR technique is the most commonly used and effective method for SARS-CoV-2 detection; however, this test often leads to population aggregation with the risk of causing virus transmission. Therefore, RT-PCR-based mixed-sample mass screening to control the COVID-19 pandemic is not optimal.

#### 2.1.2 Reverse transcription digital PCR (RT-dPCR)

In comparison with RT-PCR, RT-dPCR is a more efficient and sensitive method for SARS-CoV-2 detection.^95,96^ In recent years, RT-dPCR has been developed rapidly for nucleic acid detection and widely used in clinical microbiology.^97–99^ The principle of RT-dPCR is to split a sample into tens to tens of thousands of micro-drop units, each containing one or more copies of the nucleic acid molecule (*i.e.*, DNA template). Subsequently, each unit will amplify the target molecule, and then the fluorescence signal is counted and calculated for each unit (Fig. 2).^100–102^ Compared with RT-PCR, RT-dPCR can segment the sample to achieve non-biased target sequence amplification. In addition, RT-dPCR also facilitates the absolute quantification of the target gene copy number without calibration curves by using Poisson's statistical principle.^103,104^ Although current diagnoses mainly focus on qualitative results (positive or negative), quantitative testing of the viral copy number is essential for long-term monitoring of patient recovery or assessing the performance of a drug or vaccine.^105,106^

Recently, COVID-19 patients have been reported to revert to SARS-CoV-2 positivity;^73,107,108^ however, there are no specific clinical features to distinguish them from fully recovered patients.^86,109^ This has provoked public concern about the current standards for patient discharge.^110^ Therefore, this situation requires more sensitive detection methods compared to RT-PCR. In this case, RT-dPCR is a suitable technique with good sensitivity. Lu and team found that the LOD of RT-dPCR was 10-fold lower than that of RT-PCR.^111^ Alteri *et al.* used RT-dPCR to detect 55 suspected COVID-19 patients who tested negative by RT-PCR; however, 35% of these individuals tested positive by RT-dPCR for the N gene.^112^ Given that RT-dPCR is more sensitive in detecting the virus in low concentrations, it is a promising and reliable method to examine asymptomatic carriers discharged from hospitals. In addition, RT-dPCR can also be used to detect SARS-CoV-2 RNA in the air.^113^ It was reported that some toilets used by medical workers and patients contained a high viral load.^93,114^ This also indicates the importance of hygienic treatment and room ventilation to restrict COVID-19 transmission.

### 2.2 Isothermal nucleic acid amplification

PCR-based nucleic acid testing protocols are complicated, which require expensive instrumentation and professionals. In addition, these methods require multiple temperature changes for each cycle and involve complex thermo-cycling equipment.^115,116^ Isothermal nucleic acid amplification is another detection strategy that allows amplification without a thermal cycler at thermostatic temperature.

#### 2.2.1 Reverse transcription loop-mediated isothermal amplification (RT-LAMP)

**Fig. 3:**
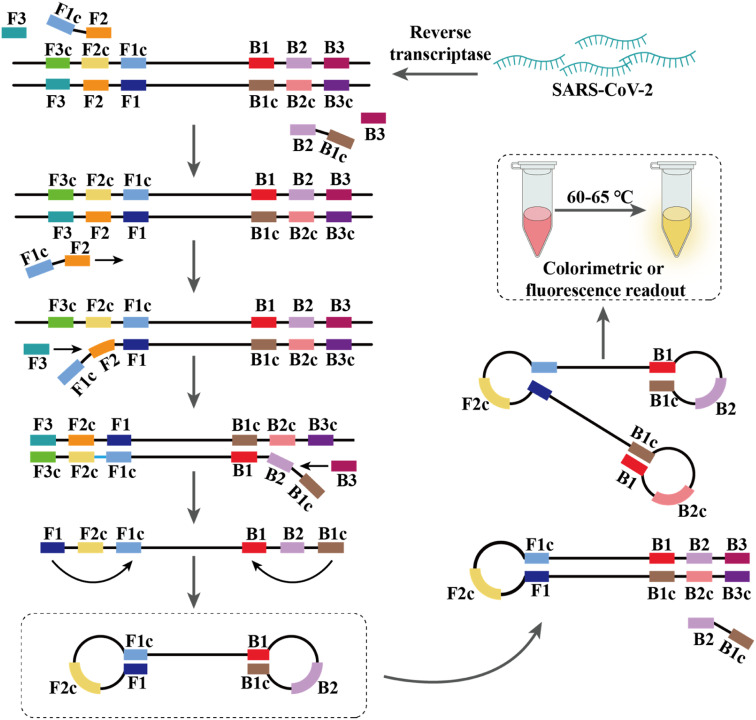
Amplification of nucleic acids using RT-LAMP. Overall, there are four core primers that mediate all the processes in a LAMP reaction by recognizing six distinct regions of the target DNA through several steps.

RT-LAMP is a high-specificity assay that uses DNA polymerase, reverse transcriptase, and 4–6 primers to bind different target regions of the genome, enabling efficient, rapid, and specific exponential amplification of target genes in less than 1 h at an isothermal condition of 60–65 °C (Fig. 3).^117–119^ Compared to RT-PCR, RT-LAMP had 100% sensitivity (95% confidence interval 92.3–100%), 100% specificity (95% confidence interval 93.7–100%), and an average time of 26.28 min for the entire reaction to detect SARS-CoV-2.^120^ Moreover, RT-LAMP also showed good accuracy (89.9–100%) in several studies.^121–124^ Yu *et al.* used an RT-LAMP method called isothermal LAMP-based method for COVID-19 (iLACO) to detect the SARS-CoV-2 ORF1ab gene and found an LOD of 10 copies per μL.^96^ Minami *et al.* evaluated a commercial RT-LAMP kit (Loopamp® 2019-SARS-CoV-2), which showed higher sensitivity with an LOD of 1 copy per μL in 35 min.^125^

In addition, many modified RT-LAMP procedures have been developed. For example, Zhang *et al.* combined the clustered regularly interspaced short palindromic repeat (CRISPR)/associated endonuclease (Cas) 12a system with RT-LAMP, and simultaneously achieved visual detection by using gold nanoparticles (AuNPs).^126^ Moreover, this method could be directly applied to 96-well plates for high-throughput screening.^127^ Thi *et al.* developed LAMP sequencing using barcode primers, which is scalable and can potentially analyze thousands of samples in parallel.^128^ Rohaim *et al.* introduced artificial intelligence (AI) algorithms in RT-LAMP and developed a handheld intelligent diagnostic device called AI-LAMP, which not only improved the performance of RT-LAMP assays but also reduced their operation time and subjectivity.^129^

#### 2.2.2 Transcription-mediated amplification (TMA)

**Fig. 4:**
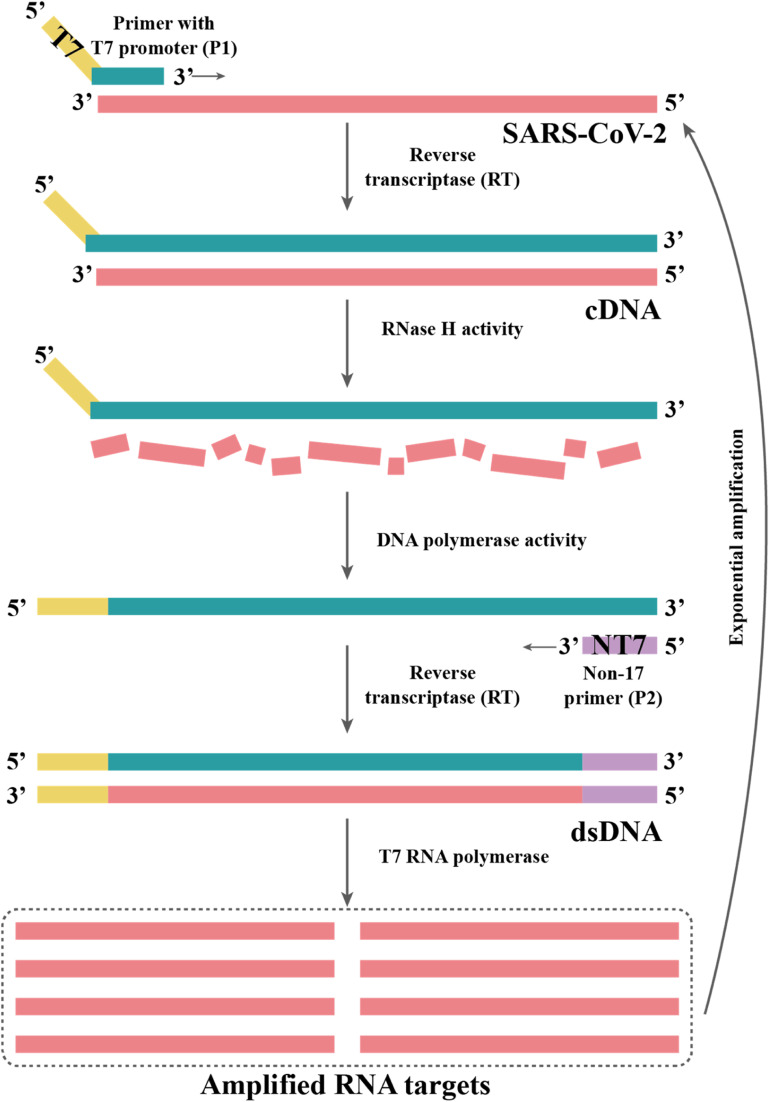
TMA begins with primers targeting an RNA region of interest, one of which contains a promoter sequence for T7 RNA polymerase. The subsequent single-stranded RNA is reverse-transcribed to cDNA by an RT in the reaction. The RNase H activity of the RT degrades the RNA in the DNA-RNA hybrid as it synthesizes the cDNA strand. This dsDNA template is transcribed to RNA by T7 RNA polymerase, resulting in the exponential amplification of the RNA target.

TMA is a technique for the direct isothermal amplification of RNA templates, which uses three enzymes including viral reverse transcriptase, T7 RNA polymerase and ribonuclease H (RNAse H).^130^ During amplification, the viral RNA is first bound to a T7 promoter primer, and then reverse transcribed into cDNA. Subsequently, the target RNA strand is degraded by RNAse H, leaving a single-stranded cDNA containing the T7 promoter. Another primer uses this single-stranded cDNA as a template to generate double-stranded DNA (dsDNA). Then, T7 RNA polymerase transcribes the generated dsDNA into RNA. Consequently, the transcribed RNA serves as the template to restart the process (Fig. 4).^131^ TMA can produce 100–1000 copies of RNA in one cycle, resulting in a 10^10^-fold increase in the target RNA in 15–30 min.^132^ Thus, TMA is popular in clinical diagnostics. Currently, many commercial tests based on this technique are available. The Aptima® SARS-CoV-2 assay is based on TMA technology that enables the detection of SARS-CoV-2, which is performed on the Hologic Panther system, a highly automated, high-throughput instrument.^109^ This assay involves the isolation of SARS-CoV-2 RNA from the sample by oligomer-coupled magnetic particles, amplification by TMA, and detection of the amplified product by chemiluminescent probes. The process takes approximately 3 h and can process over 1000 samples in 24 h.^109^ Moreover, several studies have demonstrated that the Aptima® SARS-CoV-2 assay is comparable to RT-PCR in terms of sensitivity and has good analytical performance in detecting pooled samples.^133–137^ Given this, the US Food and Drug Administration (FDA) has broadened the applicability of Aptima® SARS-CoV-2 by allowing this procedure to be used to detect asymptomatic individuals and pooled testing of symptomatic patient samples.^114^

#### 2.2.3 Reverse transcription-recombinase polymerase amplification (RT-RPA)

**Fig. 5:**
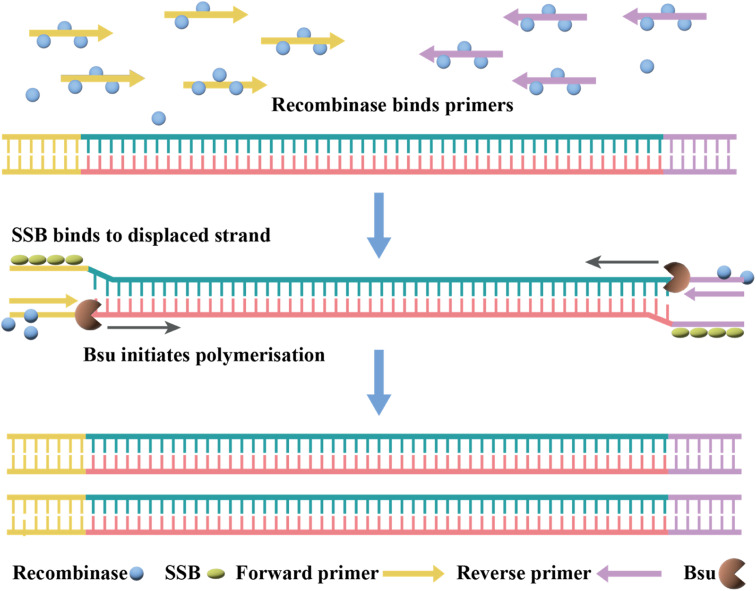
Mechanism of RT-RPA. The RT-RPA reactions typically occur between 37 °C and 42 °C in the following steps. The reaction is initiated by the binding of a recombinase (*e.g.*, T4 UvsX) and a loading factor (*e.g.*, T4 UvsY) to each of the forward and reverse primers.

RPA is considered as an alternative nucleic acid detection technique to PCR,^138^ which is capable of realizing single-molecule nucleic acid detection at room temperature within 15 min without hardware equipment.^139^ The addition of reverse transcriptase to the RPA reaction components enables the detection of RNA viruses in a single tube. Thus, this technology has been widely used to detect many RNA viruses, such as Ebola virus, Zika virus and Middle East respiratory syndrome coronavirus (MERS-CoV).^140–142^ During the reaction, the recombinase and primer junctions form protein-DNA complexes, which can search for homologous sequences in the reverse-transcribed dsDNA. Once the primer locates the homologous sequence, a strand exchange reaction occurs to form and initiate DNA synthesis and exponential amplification of the target region on the template. The replaced DNA strand binds to the single-strand binding (SSB) protein to prevent further substitution (Fig. 5).

Currently, RT-RPA can be combined with other techniques such as fluorescence resonance energy transfer (FRET) or CRISPR-Cas technology.^143–146^ El Wahed *et al.* developed RT-RPA suitcase laboratory, a device that enables COVID-19 detection in resource-poor settings. The sensitivity of RdRP, E and N gene detection for SARS-CoV-2 can reach 2, 15, and 15 RNA molecules, respectively, in 15 min.^147^ A customized isothermal amplification integrated lateral flow strip (LFS) platform enabled rapid, simultaneous visual screening of SARS-CoV-2 and influenza viruses (influenza A and influenza B) without cross-reactivity, false positives and false negatives.^148^ The other applications of RT-RPA for SARS-CoV-2 detection involve CRISPR-Cas technology, which are described in the following section.

#### 2.2.4 Other isothermal nucleic acid amplification methods

In addition to the above-mentioned isothermal amplification techniques, nicking enzyme-assisted reaction (NEAR), nuclear acid sequence-based amplification (NASBA), strand displacement amplification (SDA) and other isothermal amplification methods have been used to detect SARS-CoV-2. Herein, each technique is not described in detail, but the composition and characteristics of each isothermal amplification method are listed in Table 2.

**Table 2:** General characteristics of isothermal amplification assay for SARS-CoV-2 detection

Method	Required enzyme	Probes number	Temperature (°C)	Time (min)	Target	LOD (copies per μL)	Sensitivity	Advantages^a^	Disadvantages^a^	Ref.
NEAR	Nicking endonuclease, strand-displacing DNA	2	60–65	5–15	RdRp gene	0.125	48–71.7%	Fast testing, universally in various environments	Low sensitivity	149
NASBA	RNase H, reverse transcriptase, T7 DNA-dependent RNA polymerase	2	41	90–120	S gene N gene	0.5	98.15–100%	High sensitivity	Required RNase inhibitors	150
Non-enzymatic isothermal strand displacement and amplification	Restriction endonucleases, strand-substituting DNA polymerases	3	42	<30	RdRp gene, N gene	10	96.77%	No RNA reverse transcription step, fast testing	Non-specific reactions/false positives	151
Rolling circle amplification	DNA ligase, DNA polymerase	8	30–37	<120	N gene S gene	1	—	High specificity	False negatives and false positives	5

a Advantages and disadvantages are compared to RT-qPCR methods.

### 2.3 CRISPR-based diagnosis

**Fig. 6:**
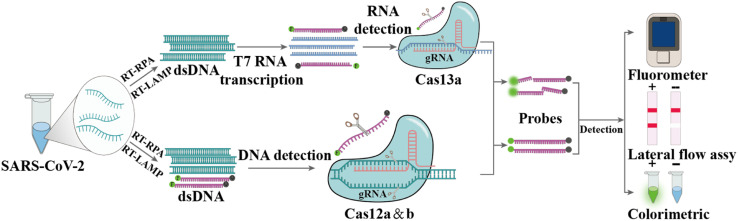
Principle of CRISPR-Cas technology for viral RNA detection. Firstly, the viral RNA is subjected to reverse-transcription and amplification, *e.g.*, in an RT-RPA reaction at 37 °C to 42 °C, to generate dsDNA. The dsDNA can be targeted by guide RNAs (gRNAs) directly in the CRISPR-Cas12 detection system, whereas RNA detection using the CRISPR-Cas13 system requires an additional T7 transcription step. When Cas12 or Cas13 is activated by the recognition of gRNA, there will be cleavage of the target as well as nonspecific cleavage of dually labeled oligonucleotide probes.

CRISPR and CRISPR-Cas are prokaryotic defense systems that safeguard organisms against exogenous nucleic acids.^152,153^ After specific binding between guide RNA (gRNA) or CRISPR RNA (crRNA) and the target sequence (DNA or RNA) located next to a proto-spacer adjacent motif, the Cas enzyme is activated, which can exhibit local DNase or RNase activity, leading to local cleavage of the target DNA or RNA (*cis*-cleavage) as well as to collateral damage to adjacent single-stranded DNA (ssDNA) or RNA (trans cleavage) (Fig. 6), respectively.^154,155^ Nowadays, many different Cas enzymes have been identified, such as Cas9, Cas12, and Cas13, in which certain enzymes can be programmed for the detection of RNA viruses.^156–158^ The Cas12a-based system DNA endonuclease-targeted CRISPR trans reporter (DETECTR) and Cas13-based system called Specific High sensitivity Enzymatic Reporter UnLock (SHERLOCK) are two representative CRISPR technologies for infection pathogen detection.^159–161^ Since the COVID-19 outbreak, the SHERLOCK and DETECTR platforms have been used for SARS-CoV-2 detection and authorized for Emergency Use Authorization (EUA) by the FDA.^162,163^

Patchsung *et al.* performed a two-step CRISPR-Cas13a-based SHERLOCK assay targeting the S, N and ORF1ab genes of SARS-CoV-2. The target sequences were reverse transcribed and amplified using RT-RPA, followed by transcribing into RNA using T7 RNA polymerase, and then identifying and detecting based on CRISPR-Cas13a with an LOD of 42 copies per reaction (Fig. 6).^164^ Broughton *et al.* developed and validated the DETECTR platform for detecting the N and S genes of SARS-CoV-2, which used RT-LAMP to amplify target genes, followed by Cas12a-based specific sequence cleavage. The resultant readout is also available by fluorescence or lateral flow. The estimated LOD of this platform is 10 copies per μL, with 95% and 100% agreement for positive and negative predictions, respectively.^165^ To fulfill COVID-19 diagnostic demands, researchers have developed many variants of CRISPR-Cas12 and CRISPR-Cas13 systems, for example, the one-pot assay iSCAN and STOPCovid.v2 (simplifying the operational steps and shortening the detection time),^166–168^ AIOD-CRISPR (avoiding aerosol contamination from opening the cap),^169^ CRISPR-FDS with the high-throughput format,^170^ ITP-CRISPR with point-of-care testing (POCT) potential in combination with microfluidics,^171^ SHERLOCK-HUDSON for direct sample detection without nucleic acid extraction^172^ and SHINEv.2 for the detection of multiple variants of SARS-CoV-2.^173^

### 2.4 Sequencing-based technology

**Fig. 7:**
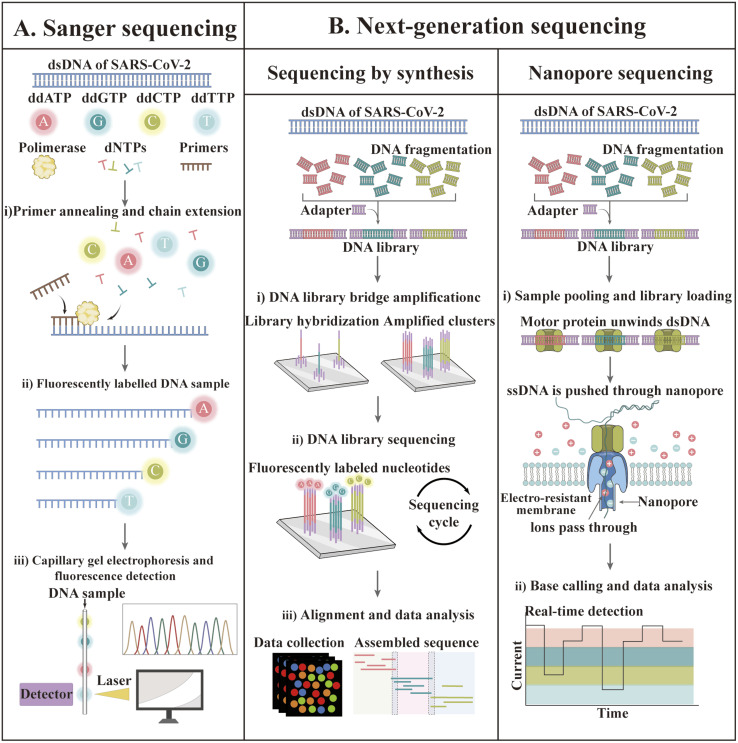
Sequencing techniques for the identification of SARS-CoV-2. (A) Sanger sequencing. Firstly, SARS-CoV-2 RNA is often amplified by RT-PCR (not depicted). Sanger sequencing reactions can be undertaken to analyze either of the DNA strands, but only one strand per reaction can be assessed. (B) NGS. Firstly, a library of millions of DNA fragments is created from a template (or enhanced by multiplex RT-PCR for SARS-CoV-2). Adapters are bound to the two ends of each DNA fragment. The adapters have a universal primer-binding site and a unique sequence (*i.e.*, barcode) that can be hybridized to a specific sequence on the support.

In recent decades, gene sequencing technology has witnessed unprecedented developments based on Sanger sequencing technology. The first SARS-CoV-2 genome was obtained by next-generation sequencing methods in less than one month after disease identification.^174^ Currently, GISAID shares more than 13 million SARS-CoV-2 genome sequences.^175^ The available sequencing technologies are Sanger sequencing as well as next-generation sequencing (NGS) technologies such as synthetic by sequencing (SBS), ion semiconductor sequencing, nanosphere sequencing, and nanopore sequencing.^176–179^ The principles of some of these techniques are illustrated in Fig. 7.

Although most SARS-CoV-2 genome sequencing is performed by NGS, traditional Sanger sequencing can still be used for viral genome sequencing.^180–183^ qSanger-COVID-19, a rapid detection kit for COVID-19 developed by BillionToOne (BTO), is based on Sanger sequencing technology, which allows direct sample detection without nucleic acid extraction and offers comparable sensitivity to RT-PCR (10–20 genomic copy equivalents); nevertheless, the throughput became 30-times faster and 20-times higher.^184^ Despite the fact that the sample processing step is eliminated, this protocol is still highly manual given that it requires setting up multiple reactions. In addition, the final sequence comparison of sequencing results must be manually checked and identified by a trained professional, making post-sequencing analysis a challenge. Unlike Sanger sequencing, which is designed to generate consistent sequences for a single target amplicon, NGS technology allows the sequencing of millions to billions of DNA strands in parallel during a single run.^185,186^ EUA has licensed four kits for the targeted testing of SARS-CoV-2 RNA based on Illumina's sequencing by synthesis technology.^161^ The details of these kits are summarized in Table 3.

**Table 3:** Some authorized sequencing diagnostic tests for SARS-CoV-2

Diagnostic	Entity	Authorized setting	Attributes	Sequencing platform	Detection target	Duration	Throughput	LOD (copies per mL)
Illumina COVIDSeq Test	Illumina, Inc.	H^a^	SBS	NovaSeq 6000	98 target genes	12 h	3072	500
Helix COVID-19 NGS Test	Helix OpCo LLC (dba Helix)	H^a^	SBS	NovaSeq 6000	S and RPP30	—	384	125
UCLA SwabSeq COVID-19 Diagnostic Platform	The University of California, Los Angeles (UCLA)	H^a^	SBS	Illumina MiSeq	S2, synthetic internal control S2 gene and RPP30	—	384	250
SARS-CoV-2 NGS Assay	Twist Bioscience Corporation	H^a^	SBS	Illumina NextSeq 550	Target the entire viral genome	—	96	800

a H, Laboratories certified under the Clinical Laboratory Improvement Amendments of 1988 (CLIA), 42 U.S.C. §263a, that meet requirements to perform high complexity tests.

To date, only a few sequencing studies have been explored for SARS-CoV-2 detection compared to the frequently used assays such as RT-PCR.^184,187–190^ Bhoyar sequenced 752 replicate processed clinical specimens using Illumina's COVIDSeq method, which shows good sensitivity, precision and accuracy compared to RT-PCR. Moreover, the diagnostic positivity of COVIDSeq increased by 5.7% (27 positives were detected from cases diagnosed as negative and indeterminate by RT-PCR).^189^ Bloom *et al.* also compared NGS (SwabSeq) with RT-PCR and found that this method had similar or better sensitivity and specificity.^191^ In addition, this SwabSeq method also has potential to detect 10 000 samples simultaneously.^191^

The Clear Dx SARS-CoV-2 test (Clear Labs) is the only EUA-authorized protocol for detecting SARS-CoV-2 using Oxford Nanopore sequencing technology. Compared to the short read length of SBS technology, Oxford Nanopore sequencing yielded longer read lengths without amplification artifacts and bias at a lower cost.^192,193^ Wang *et al.* performed nanopore target sequencing of SARS-CoV-2 and other pathogens from respiratory specimens within 6–10 h. This technique displayed considerable sensitivity and specificity, with an LOD of 10 copies per mL.^194^

## 3. Serological antibody test

Diverse antibody assays, characterized by high-throughput and low workload are playing a more crucial role in supplementing the nucleic acid test. For patients who are highly suspected of COVID-19 but negative by the molecular test, serological antibody testing may be helpful for their diagnosis.^195^ Moreover, antibody tests are essential to diagnose whether an individual has been previously infected and may also help to confirm the presence of current infection, given that a large proportion of SARS-CoV-2 infections is asymptomatic.^196^ Due to the importance of serology testing, academia and industry have developed various platforms for serological diagnosis. These tests can be used in laboratories or wherever the patient is in the hospital or at home (point-of-care). Many immunoassays, referred to as total antibody assays, have been constructed to detect levels of all isotypes simultaneously.

### 3.1 Antibody types and dynamics

**Fig. 8:**
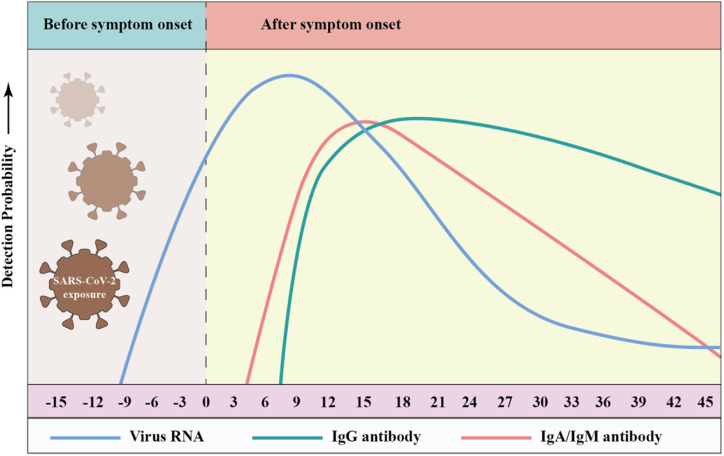
Detection probability of viral RNA or antibody (IgA, IgM, and IgG) against SARS-CoV-2 during infection (relative to symptom onset). The testing windows of nucleic acid amplification tests (RT-PCR, blue) and serology tests (antibody, green) are indicated. Reproduced with permission from *Lancet Respir. Med.*, 2020, **8**, 717–725, 10.1016/S2213-2600(20)30230-7.^211^

Antibodies are formed by the body's immune system in response to infections, which can be detected in whole blood, plasma or serum. Three types of antibodies are created in response to infection, *i.e.*, immunoglobulin A (IgA), IgG and IgM, which rise or fall at different times after the onset of infection.^197–201^ IgG is used in most antibody tests given that it persists for the longest time and may reflect longer-term immunity, while IgM typically rises quickly with infection and declines soon after infection is cleared. The early seroconversion of IgM against SARS-CoV-2 was reported at 3–5 days, and the level can last for more than 1 month, while IgG against SARS-CoV-2 may be detected after 8 days.^202,203^ Notably, many reports on serum antibody levels in SARS-CoV-2 patients indicate that IgM expression was observed concurrently with IgG expression.^204–207^ Long *et al.* conducted a large multicenter study and found that the median seroconversion of both isotypes was recorded on day 13.^208^ Udugama *et al.* found that antibody responses to infection took days to weeks to be reliably detectable. Then, the levels of those antibodies decreased over time.^209^ Although IgA has rarely been studied, as a sensitive marker of infection, IgA levels correlate with disease severity and neutralizing activity. Okba *et al.* used a commercial enzyme-linked immunosorbent assay (ELISA) to detect IgA and IgG and found that the specificity of the IgG and IgA assays were similar, but the IgA ELISA had a superior sensitivity over IgG ELISA. Fig. 8 shows that the RT-PCR molecular diagnostics as well as IgM and IgG antibody assays may vary over time.^210^

Thus far, the Foundation for Innovative Diagnostics (FIND) has listed more than 400 antibody tests, most of which are produced by commercial companies and are available on the market. Here, these assays are classified into the following categories and their properties are summarized according to the testing platforms (Table 4). It is critical to understand the strengths and limitations of these assays in various clinical and research scenarios before utilizing antibody testing for SARS-CoV-2.

**Table 4:** Comparison of the three types of SARS-CoV-2 antibody assay diagnostic kits

Method	Developer	Test	Sensitivity (%)			Specificity (%)			Antigen	Antibody	Time (min)	Specimen and volume	Advantages	Disadvantages
			IgG	TA^a^	IgM	IgG	TA	IgM						
LFIA	PRIMA Lab SA	COVID-19 IgG/IgM Rapid Test		95.7%			97.8%		NP	IgG, IgM	10	Whole blood, serum and plasma, 10 μL	Easy, fast, flexible, and accurate testing with low cost for POC testing	False positives, qualitative non-quantitative, heterogeneous presentation, the limited overall sensitivity of qualitative results only in the acute phase of the disease
	Prognosis Biotech	Rapid Test 2019-Ncov Total Immunoglobulins		98.7%			94.8%		NP	IgG, IgM, IgA	10	Serum, plasma or whole blood, 10 μL		
	Sugentech	SGTi-flex COVID-19 IgM/IgG	90%	94.4%	95%	9%	98.3%	100%	NP and S-RBD	IgG, IgM	10	Serum, plasma or whole blood, 10 μL		
	SG Diagnostics Pte Ltd	SG Diagnostics COVID-19 Immunity Rapid Test Kit	96.4%			98%			S-RBD	IgG	10	Serum, plasma or whole blood, 10 μL		
	Zhongshan Bio-Tech Co Ltd	SARS-CoV-2 IgM-IgG (GICA)	96.7%	96.7%	50%	85.0%	83.8%	97.5%	SP	IgG, IgM	10	Serum, plasma, or whole blood, 10 μL		
	Self-diagnostics	SARS CoV-2 IgM/IgG Antibody Assay Kit		88.0%			99.0%		Antigen	IgG, IgM	15	Serum, plasma, whole blood, or fingertip blood, 10 μL		
	PRIME4DIA Co., Ltd	P4DETECT COVID-19 IGM/IGG		97.7%			98.1%		NM^b^	IgG, IgM	10	Serum, plasma, whole blood, 10 μL		
	Nirmidas Biotech, Inc	MidaSpot™ COVID-19 Antibody Combo Detection Kit	96.7%	100%	100%	97.5%	100%	98.8%	NM^b^	IgG, IgM	10	Human serum, plasma, 10 μL		
	Beijing Wantai Biological Pharmacy Enterprise Co., Ltd	WANTAI SARS-Cov-2 Ab Rapid Test		100%			98.8%		S-RBD	IgG, IgM	10	Serum, plasma, whole blood, 10 μL		
	Biocan Diagnostics Inc	Novel Coronavirus (COVID-19) IgG/IgM Antibody Test		92%			100%		Antigen	IgG, IgM	10	Serum, plasma, and whole blood, 10 μL		
ELSA	Plexense, Inc.	ACCEL ELISA® COVID-19 Kit		94.4%			100%		NP	IgG, IgM, IgA	45	Blood (serum), 50 μL	Less time-consuming, low sample consumption, some analyses yield quantitative results, higher overall sensitivity compared to LFIA, suitable for high throughput and automation	Not suitable for rapid testing, requires trained laboratory personnel to check in batches during the laboratory process, endogenous interference, poor repeatability
	Quansys Biosciences	Q-Plex SARS-CoV-2 Human IgG (4-Plex)	100%			100%			SP	IgG	120	Human serum, plasma, 2 μL		
	AB Diagnostic Systems GmbH	Abia SARS-CoV-2 IgG/IgM		40.9%∼100%			91.1% ∼100%		Antigen	IgG, IgM	120	Serum or plasma		
	Actim Oy	Actim ® ELISA SARS-CoV-2 IgG	96%			98%			S1 protein	IgG	120	Serum or plasma		
	AMEDA Labordiagnostik GmbH	AMP ELISA Test SARS-CoV-2 Ab	95.9%	100%	100%	98.4%	99.5%	99.8%	NP, S1-RBD and S2-ECD^c^	IgG, IgM	70	Serum, plasma, 100 μL		
	Adversis Pharma GmbH	SARS-CoV-2 IgG ELISA	100%			99.4%			NP	IgG	120	Serum, plasma, 100 μL		
	Prognosis Biotech	Bio-Shield 2019-nCoV Total Immunoglobulins	97.3%	98.6%	82.6%	97.4%	100%	92.3%	NM^b^	IgG, IgM, IgA	120	Serum, plasma		
	Diatheva	COVID-19 ELISA IgG DIATHEVA Kit	98.8%			98.2%			NM	IgG	150	Serum, plasma		
	DIA.PRO Diagnostic Bioprobes S.r.l.	COVID-19 Spike 1&2 IgG – ELISA	98%			98%			S1 and S2 protein	IgG	120	Serum, plasma		
	Icosagen AS	Serological COVID-19 ELISA Kit		87.8%			98.9%		SP and NP	IgG, IgM	120	Serum, plasma		
CLIA	Beckman Coulter Inc.	Access SARS-CoV-2 IgG		100%			99.8%		S1-RBD	IgG	60	Serum or plasma, 20 μL	Simple operation, high sensitivity, suitable for large population detection	Expensive instruments, and well-trained professionals
	Snibe Co., Ltd	MAGLUMI 2019-nCoV IgM/IgG	92.1%	93.9%	75.7%	96%			SP and NP	IgG, IgM	60–120	Serum, 160 μL		
	Mindray	SARS-CoV-2 IgG(CLIA)	82.2%			94.9%			NM	IgG	60–120	Serum, 10 μL		
	Inova Diagnostics, Inc.	QUANTA Flash® SARS-CoV-2 IgG	100% (15+ days)			99.9%			SP or NP	IgG	60–120	Serum or plasma, 20 μL		
	Ortho Clinical Diagnostics	VITROS Immunodiagnostic Products Anti-SARS-CoV-2 Total Reagent Pack		100% (8+ days)			100%		S1 protein	IgG, IgA, and IgM	∼85	Serum or plasma, 80 μL		
	Immunodiagnostic Systems Ltd	IDS SARS-CoV-2 IgG	97.6% (15+ days)			100%			NP and SP	IgG	About 60 ∼ 120	Serum or plasma		
	Beckman Coulter	Access SARS-CoV-2 IgM			98.3%(15+ days)			99.9%	S-RBD	IgM	About 60 ∼ 120	Serum or plasma, 10 μL		
	Siemens Healthcare Diagnostics Inc.	Dimension Vista SARS-CoV-2 Total Antibody Assay (COV2T)		100% (15+ days)			99.8%		S-RBD	IgG, IgM	About 60 ∼ 120	Serum or plasma, 10 μL		
	Ortho-Clinical Diagnostics, Inc.	VITROS Immunodiagnostic Products Anti-SARS-CoV-2 IgG Reagent Pack	100% (15 days+)			100%			SP	IgG	About 85	Serum or plasma, 20 μL		
	DiaSorin Inc.	LIAISON® SARS-CoV-2 S1/S2 IgG	97% (15+ days)			98%			S1 and S2	IgG		Serum or plasma, 20 μL		

a TA, total antibodies.

b NM, not mentioned.

c ECD, extracellular domain.

### 3.2 Lateral flow immunoassay (LFIA)

**Fig. 9:**
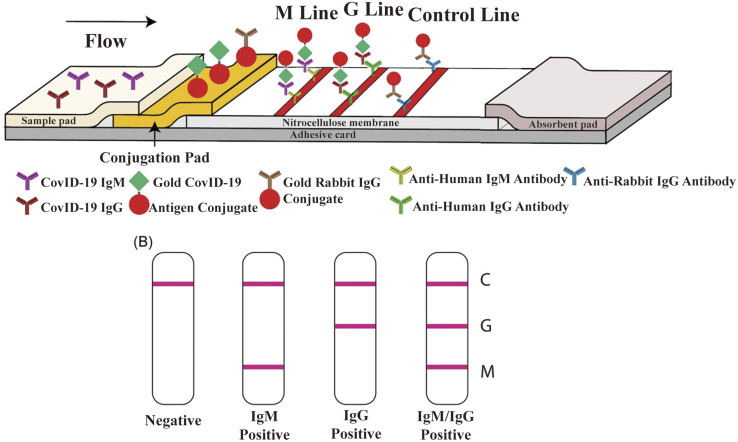
Schematic illustration of rapid SARS-CoV-2 IgM-IgG combined antibody test. (A) Schematic diagram of the detection device. (B) Illustration of different testing results, C: control line; G: IgG line; and M: IgM line. Reproduced with permission from *J. Med. Virol.*, 2020, **92**, 1518–1524, 10.1002/jmv.25727.^224^

LFIA is a rapid immunochromatography-based method that utilizes a cassette to inject patient samples.^212–215^ Usually, it requires only a few drops of whole blood from a finger prick placed on the test strip, with a band appearing as positive or negative for antibody detection. If the sample contains SARS-CoV-2-specific antibodies, they will attach to the virus antigen bound to AuNPs or another detection system, where the complex migrates along the membrane towards the test line containing a secondary antibody against the immune complex to result in a visible band.^216^ LFIA is a POC serology test, which can be used in emergencies with the advantages of time-saving (∼15 min), easy-operation, and easy-result-interpretation. Guedez-López *et al.* evaluated three commercially available antibody detection kits and found that their sensitivities were relatively low in the early stage (1–7 days from the onset of symptoms) of SARS-CoV-2 infection and gradually increased in the intermediate stage (8–14 days from the onset of symptoms) and peaked at a late stage (more than 15 days).^217^ After systematic analysis of 5016 references and 40 studies, Bastos *et al.* also concluded that LFIA had lower sensitivity in the early stage of symptom onset.^218^ Although the sensitivity of the assays was affected by the timing of sample acquisition, a higher overall sensitivity was consistently observed with the use of total antibody detection. Li *et al.* developed an LFIA for the simultaneous detection of IgG and IgM and found a significant increase in sensitivity compared to the detection of IgG or IgM, respectively (Fig. 9).^219^ Other studies reported the sensitivity and specificity of LFIA for SARS-CoV-2-specific total antibodies using recombinant antigens of NPs, which were 94.6% (84.9–98.9, CI 95%) and 100% (95.75–100, CI 95%) as early as 7 days post confirmation of positivity, respectively.^220^ In addition, an immunochromatographic fluorescence assay was developed by combining fluorescent nanotags with LFIA to ensure high sensitivity and reliability of the assay. These fluorescent nanotags such as silica-core@dual QD-shell nanocomposites (SiO_2_@DQD),^221^ fluorescent microspheres^222^ and selenium nanoparticles^206^ showed excellent accuracy when labeled with NP or SP of SARS-CoV-2. Notably, Chen *et al.* designed aggregation-induced emission (AIE) nanoparticles (emission wavelength, 810 nm)-labeled LFIA for the early detection of antibodies against SARS-CoV-2 and the sensitivity of the proposed test strip for detecting IgM and IgG was 78% and 95%, respectively (172 serum samples).^223^ Importantly, the detection of IgM or IgG in sequential clinical samples was 1–7 days after symptom onset. Recently, Liu and coworkers developed a surface-enhanced Raman scattering (SERS)-based LFIA biosensor,^221^ which used dual-layer Raman molecule-loaded Ag-coated SiO_2_ nanoparticles as advanced SERS tags. Subsequently, the SERS tag was conjugated with the S protein for the simultaneous detection of IgG/IgM antibodies with the LOD of 1 ng mL^−1^ S^−1^-protein antibody.^221^ Further the LOD was 1 pg mL^−1^ for clinical samples, which is 1000-times lower than the visualization results. Thus, the SERS-LFIA technique was proposed for rapid screening and bulk diagnosis at ultra-low detection levels when other commonly used methods are not available.

### 3.3 Indirect enzyme-linked immunosorbent assay

Indirect ELISA is a plate-based assay, in which the microtiter wells are coated with antigens. After adding the sample, the antibodies will specifically recognize the immobilized antigens. After washing, a secondary antibody conjugate is introduced, which specifically binds to the antigen/antibody complex. Then, the color is developed, and the absorbance is related to the number of antibodies in the sample.^225–227^ Indirect ELISA is easily adaptable to automation for high throughput. Most indirect ELISAs utilize N or S proteins alone or in combination with antigens from the ORF1ab region. Okba *et al.* produced an array of different ELISA to examine antibody reactivity to SP, S1, and NP proteins, and S-RBD antigens^210^ and found that the sensitivity of the SP-based ELISA is higher than that of the S1-based ELISA. However, the specificity of S1-based ELISA is higher, because SP-based ELISA is cross-reactive with SARS-CoV and MERS-CoV. In a similar study, the same group found that compared to S-RBD-based ELISA, S-based ELISA is more sensitive,^228^ which can be expected considering that SP contains subunits S1 and S2. Lopandić *et al.* produced and purified recombinant protein M, which can specifically bind to antibodies from the sera of COVID-19 convalescents, and in M-based ELISA for the detection of IgG and IgM, the sensitivities were 96% and 93%, respectively.^229^ In general, all these antigens showed comparable sensitivities when assayed. Recently, numerous magnetic ELISA assays have been developed by combining magnetic bead systems with ELISA. Cai *et al.* reported that biotinylated synthetic peptides comprising different parts of SARS-CoV-2 proteins were bound to streptavidin-coated magnetic beads, providing 81.5% sensitivity for COVID-19 IgG and IgM detection in about 30 min.^230^ Following this, the authors used this method to detect cases of COVID-19 antibodies in RT-qPCR-negative infected asymptomatic patients, highlighting the importance of antibody detection. Subsequently, Huergo *et al.* immobilized recombinant His-tagged SARS-CoV-2 NP on the surface of Ni^2+^ magnetic beads and challenged whole blood samples obtained from controls or COVID-19 cases.^231^ The method only required 2 μL of whole blood, and the detection procedure could be accomplished in 12 min. Ultimately, the naked eye could evaluate the results without sophisticated instruments. Presently, indirect ELISA-based methods may offer the advantages of measuring antibody titers, high throughput and selective isotype detection, but they are labor-intensive and unsuitable for POC testing. Thus, to address these issues, microfluidic ELISA was established. Gong *et al.* developed a microfluidic platform that collected serum by a pulling-force spinning top and paper-based microfluidic ELISA for quantitative IgA/IgM/IgG measurements in an instrument-free manner.^232^ This method could isolate the serum from whole blood and provide an affordable, rapid and user-friendly way to diagnose COVID-19. Furthermore, it had higher sensitivity for detecting total antibodies compared to conventional methods (99.7% *vs.* 95.6%). Liu *et al.* solved this dilemma by presenting a reciprocating-flowing immunobinding (RF-immunobinding) strategy, which enabled the antibodies in the fluid to come into contact with the corresponding immobilized antigens on substrate repeatedly during continuous reciprocating-flowing to achieve adequate immunobinding within 60 s.^233^ This strategy was further developed into an immunoassay method for the serological detection of 13 suspected COVID-19 patients and obtained a 100% true negative and true positive.

### 3.4 Chemiluminescence immunoassay (CLIA)

CLIA offers significant advantages over traditional assay detection methods, especially in the quantification of antibodies. Its principle is that the recombinant protein of SARS-CoV-2 is labeled by magnetic beads and used to bind the antibody (IgG, IgM, or IgA) in the sample, followed by the use of a secondary antibody coupled with chemiluminescent agents to recognize the antibody. Finally, a luminous signal will be generated when the substrate is added.^234,235^ Typically, CLIA results are obtained in 0.5–2 h. Similar to ELISA, CLIA is a high-throughput assay with high accuracy, low signal-to-noise ratio, and increased stability of reagents. Many studies utilized CLIA to address the differences in patient populations and time of onset of symptoms *vs.* serologic results. Grossberg *et al.* used a multiplex CLIA test for COVID-19 in an otherwise healthy cohort of adults and children in Colorado, and found that IgM antibodies against SARS-CoV-2 were generally detectable in the blood several days after initial infection, and the IgM levels and IgG levels were both elevated early (0–30 days following symptom onset). The IgM levels declined 30 days following symptom onset, while the IgG levels remained elevated for up to 60 days following the onset of symptoms.^236^ In another study, Long *et al.* utilized CLIA to test 285 patients with COVID-19 and found that within 19 days after the onset of symptoms, 100% of patients were tested positive for IgG. Because seroconversion for IgG and IgM occurred simultaneously or sequentially, both IgG and IgM titers plateaued within 6 days after seroconversion.^237^

The quantitative index is significant as a biomarker, reflecting the severity of the clinical manifestations of patients. Kong *et al.* investigated the level of serologic IgM and IgG antibodies and compared the results of CLIA with nucleic acid test (NAT). Among 88 patients, 95.45% were confirmed as positive by the combination of NAT and CLIA, which was significantly higher than by single NAT (73.86%) or CLIA (65.91%).^238^ They also found that the seroconversion started on day 5 after disease onset, and the IgG level rose earlier than IgM. The comparison between patients with different disease severity suggests that early seroconversion and high antibody titer are linked with less severe clinical symptoms. These results support the combination of CLIA and NAT in routine COVID-19 diagnosis. It is worth noting that this result is opposite to a study that reported a positive correlation between clinical severity and antibody titer.^238^ Different quantification methods, especially the precision, range and linearity of quantification may be responsible for these differences. Besides the above-mentioned three major categories of methods, there are some other detection techniques such as fluorescent microsphere immunoassays,^239^ photonic ring immunoassays^240^ and photometric immunoassays.^241^

With the popularity of vaccination, it is increasingly accepted that serological antibody tests may serve to evaluate vaccine protectiveness. However, the concept of antibody-based immunization passports did not succeed. The FDA states that antibody testing cannot be used to determine immunity or protection against COVID-19, especially after individuals receive the COVID-19 vaccine.^242^ Although serological tests demonstrate high sensitivity and specificity, some tests detect antibodies that may be only produced after natural infection. Depending on the assay, people who were not previously infected could test negative for antibodies despite the fact that they have vaccine-induced immunity. Nowadays, antibody tests can play an important role in identifying individuals who may have been exposed to the SARS-CoV-2 virus and may have developed an adaptive immune response.

## 4. Neutralizing test

To prevent the spread of SARS-CoV-2, many countries are diligently promoting vaccination to achieve herd immunity as soon as possible. Vaccine protection efficacy is the most critical parameter in evaluating vaccine clinical effectiveness. Meanwhile, the detection of neutralizing antibodies also plays a crucial role in assessing the level of human immune response after vaccination. Antibody assays, such as SARS-CoV-2 IgG II Quant, Actim ELISA SARS-CoV-2 IgG and AMP ELISA Test SARS-CoV-2 IgG, that use spiked protein as the detection antigen will not distinguish the antibody response to vaccination from the reaction to natural viral infection. In contrast, the assays that use the N protein as a detection antigen, such as Platelia SARS-CoV-2 Total Ab, Biohit SARS-CoV-2 IgM/IgG Antibody Test Kit, and New York SARS-CoV microsphere immunoassay, may only detect the response to viral infection, but not for response to vaccination. The main constraint of all the above-mentioned antibody tests is that they all detect binding antibodies against the SARS-CoV-2 antigen, which serves only for population-based serosurveillance to understand the epidemiology of COVID-19, such as seroprevalence; however, the analysis results will not directly indicate whether an individual is immune to SARS-CoV-2 infection. The neutralization potential of antibodies in serum can only be ascertained using neutralization tests. Table 5 presents a summary of the characteristics of current neutralizing antibody (nAb) assays.

**Table 5:** Summary of current neutralization assays

nAb test method	Test time	Biosafety Level	Pros	Cons
Live virus neutralization test	3–4 days	3	Accurate, gold-standard testing conditions close to the reality	Prolonged, low security, expensive and time-consuming, complex analysis leading to high variability
Pseudovirus neutralization test	3–4 days	2	More accessible, safer, and more sensitive than PRNT	Still very slow and complicated
Surrogate virus neutralization test	2–3 h	1	Fast and easy, simple system, high throughput, high sensitivity, no virus required	Only detecting partial nAb, unable to measure fusion-blocking antibodies
Lateral flow assay for neutralization test	10–15 min	0–1	Most accessible, fastest, and easiest to use outside the lab	Similar to the surrogate virus neutralization test

### 4.1 Live virus neutralization test

**Fig. 10:**
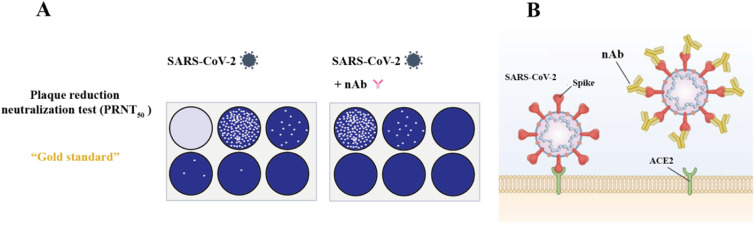
Cartoon illustrating the detection of nAb. (A) For the analysis, the sera were 2-fold serially diluted (1 : 20 to 1 : 640) and incubated with SARS-CoV-2. The PRNT titer was calculated based on a 50% reduction in plaque counts (PRNT_50_). The PRNT_50_ titer was chosen according to the WHO guideline. Reproduced with permission from *Viruses*, 2022, **14**, 410, 10.3390/v14020410.^253^ (B) When nAb is absent, spike proteins of SARS-CoV-2 interact with ACE2 receptors (ACE2) to allow viral entry, replication, and subsequent plaque formation. The presence of nAb inhibits viral entry and HEK293T is commonly expressed ACE2 together with TMPRSS2. Reproduced with permission from *J. Mol. Sci.*, 2021, **22**, 2268, 10.3390/ijms22052268.^254^

The gold standard for detecting nAb is the plaque reduction neutralization test (PRNT), which requires serial dilution of patient serum and incubation with authentic virus, followed by infection of cells (including animals, chicken embryos, and cells). After several proliferation cycles, it forms a restricted cytopathic cell area known as “plaque” (Fig. 10).^243–245^ For assays, the nAb titer was evaluated regarding 50% (PRNT_50_) or 90% (PRNT_90_) plaque reduction. PRNT seldom requires particular reagents and offers excellent sensitivity. However, it has several limitations, including high technical requirements, cumbersome operation, low throughput and difficulty in automation. Thus, to address the shortcomings of PRNT, an alternative analytical method called the focus reduction neutralization test (FRNT) was developed. FRNT is carried out in a 96-well plate and utilizes immunostaining to display infected lesions that can be counted using a computer-controlled imager, significantly increasing the analytical throughput compared to manual counting performed in PRNT.^246^ FRNT has been frequently employed to longitudinally assess the antibody response in SARS-CoV-2 infection owing to its high sensitivity and capability to quantify neutralizing antibody titers in serum.^228,247–249^ In addition, many researchers verified their experimental results using this method as a control experiment.^250–252^

In summary, for the detection of nAbs against SARS-CoV-2, live virus nAb assays are highly effective methods. However, the turnaround time required for the virus to form visible plaques is too long, and all the experimental procedures must be processed in a biosafety level 3 (BSL-3) containment facility. Hence, this approach is not feasible for large-scale serological diagnosis and vaccine evaluation.

### 4.2 Pseudovirus neutralization test

To overcome the containment of BSL-3, pseudotyped virus engineering has been developed. Pseudovirus (or pseudotyped virus) is a recombinant viral particle that expresses the SARS-CoV-2 S protein on its surface. Pseudovirus binds to ACE2, which is over-expressed by target cells and highly mimics the invasion process of SARS-CoV-2.^255–257^ To facilitate readout when designing, pseudotyped viruses typically harbor reporter genes encoding NanoLuc luciferase or green fluorescent protein (GFP).^258,259^ The selection of cell lines and viral models will influence the neutralization activity of the antibody. In most pseudovirus neutralization assays, the Vero, Huh7 and HEK293T cell lines were frequently utilized.^260–263^ Nie *et al.* infected six diverse cells (human- and animal-derived cell lines) using the developed vesicular stomatitis virus (VSV) pseudovirus and found that all the cells exhibited high susceptibility, where Huh7 cell signaling was the highest,^264^ which is consistent with the previously reported result.^265^ However, it is worth noting that SARS-CoV-2 replicates weakly in the Huh7 cell lines, as shown in some live virus neutralization assays.^266^ To enhance the infectivity of cells in pseudovirus neutralization assays, HEK293T cells are frequently modified to express both ACE2 and TMPRSS2, making them more susceptible to infection.^267,268^ VSV, human immunodeficiency virus-1 (HIV-1) and murine leukemia virus (MLV) are often chosen as vectors in the construction of pseudoviruses. Table 6 presents a summary of the SARS-CoV-2 neutralization assays that used these vectors. Among them, VSV is the most frequently utilized vector because it can be engineered into two formats, *i.e.*, a replication-deficient VSV that lacks the G protein (VSVΔG) and a replication-capable VSV/SARS-CoV-2 chimeric virus.^269–272^ Case *et al.* produced a high-titer, replication-competent chimeric VSV, which expressed the SARS-CoV-2 S protein and performed similarly to the SARS-CoV-2 clinical isolate in multiple neutralization assays.^273^ In another study, Schmidt *et al.* developed a series of SARS-CoV-2 S-pseudotype, single-cycle, HIV-1 and VSV-based methods, as well as replication-capable VSV/SARS-CoV-2 chimeric viruses for the detection of nAb. They simultaneously assessed the differences among these pseudoviruses and live viruses and found that the HIV-1 and VSV-based pseudotyped viruses were slightly less sensitive to neutralization compared to the live SARS-CoV-2, especially by weakly neutralizing plasma. In contrast, VSV/SARS-CoV-2 chimeric viruses were similarly susceptible to neutralization by monoclonal antibodies to authentic SARS-CoV-2. More interestingly, VSV/SARS-CoV-2 chimeric viruses have been shown to be more sensitive to plasma neutralization than SARS-CoV-2.^274^ Notably, the irreproducible HIV-1 and VSV-based pseudoviruses used by other researchers displayed the identical reliable performance as live SARS-CoV-2 in detecting SARS-CoV-2 nAbs^275,276^ In addition, many studies have constructed pseudoviruses by truncating the C-terminus of the S protein and introducing a D614G mutant spike to increase the infectivity titer of SARS-CoV-2 pseudotypes in neutralization experiments.^277–280^ In the assay, single-cycle pseudovirus neutralization assays allow direct readout of the percentage of virus blocked from entering in a single round of infection, and replicating chimeric viruses can be employed to assess the capability of nAb to reduce virus particle growth or eliminate the virus. Thus, the selection of cell and pseudovirus models is warranted for the assay. A reagent public repository is provided for our selection (https://www.beiresources.org/).

**Table 6:** Types of SARS-CoV-2 pseudovirus neutralizing tests

Sample type	Pseudoviral vector	Fluorescent gene	Target cell	TMPRSS2 expression	Type of SP	Effect compared with SARS-CoV-2 virus	Turnaround time	Biosafety level	Ref.
Healthy adults immunized with the mRNA vaccine	VSVΔG	Firefly Luciferase	A549	Engineered	Full-length SP, D614G mutation	Similar	∼2 days	2	281
COVID-19 seropositive samples	VSVΔG	Luciferase	Vero E6	No	Full-length SP	—	∼3 days	2	282
Convalescent patient sera	VSVΔG	Firefly luciferase	Huh7	No	Full-length SP	—	∼3 days	2	283
Serum samples from COVID-19 and health donors	VSVΔG	Luciferase	Vero E6	No	Spike-Δ19	—	∼1 day	2	284
Healthy adults immunized with the vaccine	VSVΔG	GFP and luciferase	Vero 76	No	—	Consistent	∼3 days	2	269
Germline-encoded interferon	VSV chimeric virus	GFP	HEK293	Engineered	Spike-Δ19	—	∼3 days	2	285
Human plasma samples COV-47, COV-72 and COV-107 and monoclonal antibodies C144, C135 and C121	VSV chimeric virus	GFP	HEK293T	No	Spike-Δ19	—	∼2 days	2	286
mAbs against the RBD and convalescent sera	VSV chimeric virus	eGFP	Vero E6	Engineered	Spike-Δ21	—	∼2 days	2	256
mAbs, animal immune sera, human convalescent sera, and human sera immunized by mRNA vaccine	VSV chimeric virus	Luciferase	Vero E6	Engineered	Spike-Δ19	Consistent	∼2 days	2	287
Convalescent plasma and human monoclonal antibodies	VSV chimeric virus	GFP	Vero E6	No	Spike-Δ18	Better than SARS-CoV-2	∼3 days	2	288
Rhesus macaque anti-SARS-CoV-2 serum	HIV-1	Firefly luciferase	HEK293T	No	Spike-ΔCT13	—	∼3 days	2	289
Human monoclonal antibodies	HIV-1	Firefly luciferase	HEK293T	Engineered	Full-length SP	—	∼3 days	2	290
Animal immune sera, human convalescent sera, and human sera immunized by mRNA vaccine	HIV-1	Luciferase	Vero E6	Engineered	Spike-Δ19	Inferior to SARS-CoV-2	∼3 days	2	291
Convalescent plasma and human mAbs	HIV-1	NanoLuc luciferase and GFP	Vero E6	No	Spike-Δ19	Inferior to SARS-CoV-2	∼3 days	2	288
Convalescent COVID-19 patient sera	HIV-1	Luciferase	HEK293T	No	Full-length SP	—	∼3 days	2	292
S2X259 mAb	MLV	Luciferase	Vero E6	No	Spike-Δ19	Inferior to SARS-CoV-2	∼4 days	2	293
Blood samples	MLV	Firefly luciferase	HEK293T	No	Spike-Δ19	—	∼3 days	2	294
Mouse mAb 10G6H5 against SARS-COV2 S protein, human plasma from COVID-19 patients	MLV	Luciferase	HEK293T	Engineered	Full-length SP	—	∼3 days		267

Overall, pseudoviruses are relatively safe and reliable, with similar testing results to live SARS-CoV-2 viruses, which can be used for high-throughput assays. Nevertheless, the pseudovirus has different kinetics in comparison with live SARS-CoV-2 when expressing SP, which may cause testing bias.

### 4.3 Surrogate virus neutralization test (sVNT)

Although the pseudovirus-based nAb test overcomes the limitation of BSL-3, it still requires the use of live viruses and cells. In addition, the analytical results are heterogeneous between laboratories due to the different culture conditions, virus strains and cell lines. In this case, sVNT can address the aforementioned issues.

The majority of sVNTs are based on the mechanism of blocking the interaction between RBD and ACE2 as well as the high immunogenicity of the RBD. Typically, 96-well plates are coated with the human angiotensin-converting enzyme 2 (hACE2) receptor, and then test sera are co-incubated with horseradish peroxidase (HRP)-conjugated recombinant SARS-CoV-2 RBD fragments (HRP-RBD). The binding of HRP-RBD and hACE2 is blocked by nAbs against SARS-CoV-2, and this blocking effect can be detected by reducing the HRP luminescence signal.^295–298^ Alternatively, plates can be encapsulated with RBD, soluble hACE2 for competition with nAbs.^299,300^ After comparing these two immobilization assays, Abe *et al.* found that the immobilized RBD and soluble hACE2 were more sensitive.^301^

At present, most sVNTs are ELISA-like methods. To date, many commercial kits based on this method have been developed. Krüttgen *et al.* evaluated two ELISA-based sVNT kits and discovered that they both displayed high sensitivity and specificity.^302^ Michiels *et al.* compared the sVNT commercial kit (GenScript cPass™) with a live virus neutralization assay and Luminex multiplex immunoassay (MIA), and studied two different cohorts using these three methods.^303^ The sVNT obtained a sensitivity of 94% (CI 90–96%) and 89% (CI 81–93%) compared with the other two methods. They also found that the different methods had a strong antibody titer correlation (*r*^2^ > 0.8). Interestingly, Tan *et al.* found that sVNT was as specific as the live virus neutralization assay and more sensitive than it.^304^ Besides, Bošnjak *et al.* compared sVNT with the pseudovirus neutralization test (PVNT) and found a strong positive correlation between sVNT and PVNT_50_ (*r*^2^ = 0.7135, *p* < 0.0001) as well as PVNT_90_ (*r*^2^ = 0.5042, *p* < 0.0001) inhibitory titers.^305^ Embregts *et al.* also studied 298 PCR-confirmed patient sera using sVNT and PVNT and found that sVNT had moderate to high sensitivity (91.3%) and specificity (100%), but it was not sensitive to detect low titers.^306^ However, when using a cut-off value of 50% inhibition, highly neutralized samples could be identified, which is similar to a previous report.^295^

**Fig. 11:**
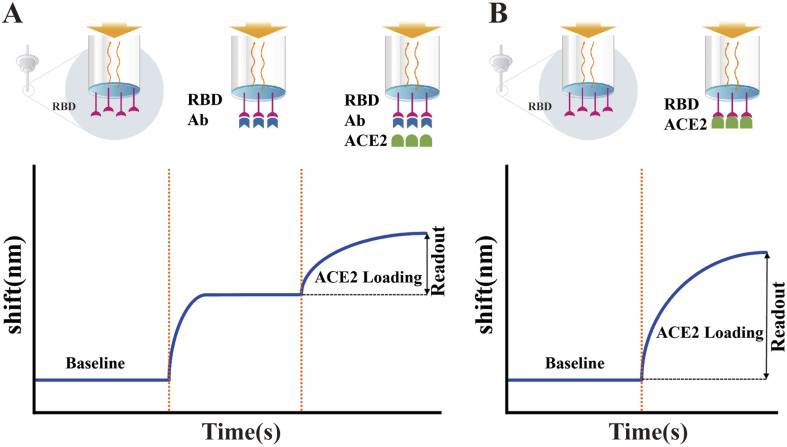
Illustration of the LF-sVNT protocol and example sensorgrams. (A) First cycle measuring the binding ability of RBD to ACE2 after neutralization. (B) Second cycle measuring the full binding ability of RBD without neutralization. Reproduced with permission from *J. Clin. Microbiol.*, 2021, **59**, e0019321, 10.1128/JCM.00193-21.^308^

In addition to the above-mentioned ELISA-based sVNTs, many other forms of sVNTs have been developed. Wang *et al.* developed a track-etched microporous membrane filtration microplate (TEM) and optical fibers transmitted immunosensing smartphone platform (TEMFIS)-based surrogate virus neutralization test (TEMFIS-sVNT) for rapid one-step testing of nAb to SARS-CoV-2.^307^ The entire assay process only took 45 min. The pore size of the TEM is 3 μm, allowing softer red blood cells, smaller platelets and fresh plasma blood components to pass through or be washed away, and thus this device can be used to assay serum, plasma and whole blood samples. In addition, TEMFIS-sVNT has a strong statistical correlation (*R*^2^ > 0.8) with ELISA-sVNT and pVNT. However, it requires HRP-tagged RBD. Thus, to address this issue, Luo *et al.* established an alternative virus neutralization test on a label-free immunoassay platform (LF-sVNT).^308^ The LF-sVNT mimics the SARS-CoV-2 surface using a sensing probe coated with RBD and simulates host cells using ACE2, which models virus-host cell interactions. Compared to other sVNTs, LF-sVNT eliminates the effects of attachment of secondary antibody-labeled enzymes,^309^ extension of streptavidin-labeled enzymes or fluorophores,^301,310^ and performing color-generating enzyme reactions.^301,304,309,311^ In addition, this platform allows real-time monitoring of RBD-ACE2 interactions to reduce the experimental error rates (Fig. 11).

Overall, sVNT is more straightforward, cheaper and faster (typically 1–2 h) than other nAb assays, making it more suitable for rapidly screening many samples. In addition, because the test is not species antibody-dependent, it can detect nAbs in any animal species used in preclinical testing of the SARS-CoV-2 vaccine. However, the drawback of sVNT is that it is unable to detect nAbs other than that bound to SARS-CoV-2 S-RBD. However, these non-RBD-targeting antibodies play only a minor role in SARS-CoV-2 neutralization.^312,313^ Nevertheless, although sVNT analysis may never completely replace conventional virus neutralization test (cVNT), the performance of sVNT is closely related to that of cVNT and pVNT.

### 4.4 Lateral flow assay (LFA)-based neutralization test

Currently, the majority of LFAs are based on the principle of antibody–antigen interaction. In general, the higher the affinity of the antibody, the higher the sensitivity of the assay. Thus, antibodies with high affinity for the antigen can also be developed with modern antibody technologies, making this type of assay relatively easy to implement. Protein–protein interaction without antibodies can also be applied in lateral flow assays. However, it is very rare, given that the binding affinity between proteins is commonly lower than that between antibodies and antigens and cannot be artificially increased. A lower binding affinity or lower analytical sensitivity often results in a longer analysis time, which requires high-sensitivity detection systems such as fluorescent labeling and fluorescent detection instruments.^27^

Fortunately, the SARS-CoV-2 S-RBD exhibits high affinity to ACE2. The lateral flow competitive protein binding assay was developed based on the interaction between RBD and ACE2 or between the S1 protein and ACE2.^312,314,315^ One of the recombinant proteins is labeled with a colored material (usually colloidal gold), and another is dispensed on a nitrocellulose membrane to form the test line. During the assay, the presence of nAb in the sample will block the interaction between the labeled-RBD and ACE2, by which the intensity of the detection line on the nitrocellulose membrane can be measured. Alternatively, the colored material labeled S1 or RBD may also influence the detection sensitivity. Zhang *et al.* compared the analytical performance of AuNPs-tagged RBD and AuNPs-tagged S1 and found that LFA using AuNPs-tagged S1 was more sensitive.^316^ However, although this finding is predictable, given that there are many reports that RBD is the main part of the S1 protein, which plays a neutralizing role, some nAbs also bind to the NTD of the S1 protein, playing a neutralizing role.^317–320^ Although the sensitivity is different using RBD or S1, nAb-LFA is as effective as the pseudovirus neutralization assay for detection.^316^

Considering the convenience, speed and simplicity of the LFA-based neutralization test, it can be used both in professional laboratories and by individuals at home. Therefore, the development of LFA-based SARS-CoV-2 nAb assays can complement the available methods for detecting nAb. Table 7 presents a summary of the commercially available LFAs for the detection of SARS-CoV-2 nAb.

**Table 7:** Commercial kit for the detection of SARS-CoV-2 nAb

Kit name	Regulatory status	Time to result (min)	Self-testing/self-collection	Validated sample types	Impact of SARS-CoV-2 variant	Sensitivity	Specificity
SARS-CoV-2 nAb Assay (colloidal gold)	CE-IVD	15	Intended for professional use only	Plasma	Alpha – unknown	97.62%	100.00%
				Serum	Beta – unknown		
				Whole blood	Gamma – unknown		
2019-nCoV nAbs Test (fluorescence immunochromatographic assay)	CE-IVD	15	Intended for professional use only	Plasma	Alpha – unknown	98.40%	98.40%
				Serum	Beta – unknown		
				Whole blood	Gamma – unknown		
SARS-CoV-2 nAb Rapid Test Device	CE-IVD	15	Intended for self-collection (kit available)	Plasma	Alpha – unknown	93.86%	99.89%
				Serum	Beta – unknown		
					Gamma – unknown		
reOpenTest™ SARS-CoV-2 nAb Rapid Test	CE-IVD	10	Intended for professional use only	Plasma	Alpha – no expected impact (*in silico* analyses)	98.82%	99.17%
				Serum	Beta – no expected impact (*in silico* analyses)		
				Whole blood	Gamma – unknown		
SARS-CoV-2 nAbs Test	CE-IVD	15	Intended for professional use only	Plasma	Alpha – unknown	98.80%	99.14%
				Serum	Beta – unknown		
				Whole blood	Gamma – unknown		
Coronavirus (SARS-CoV-2) nAb Rapid Test	CE-IVD	10	Intended for professional use only	Plasma	Alpha – no expected impact (*in silico* analyses)	95.70%	100.00%
				Serum	Beta – no expected impact (*in silico* analyses)		
				Whole blood	Gamma – unknown		
SARS-CoV-2 nAb Rapid Test	CE-IVD	15	Intended for professional use only	Plasma	Alpha – no expected impact (*in silico* analyses)	98.51%	99.50%
				Serum	Beta – no expected impact (*in silico* analyses)		
				Whole blood	Gamma – unknown		
SARS-CoV-2 nAb Test Kit (fluorescence immunoassay)	CE-IVD	15	Intended for professional use only	Plasma	Alpha – unknown	95.06%	99.00%
				Serum	Beta – unknown		
					Gamma – unknown		
SARS-CoV-2 nAb TestKit (colloidal gold chromatographic immunoassay)	CE-IVD	15	Intended for professional use only	Plasma	Alpha – unknown	96.50%	100.00%
				Serum	Beta – unknown		
				Venous blood	Gamma – unknown		
V-Pass SARS-CoV-2 nAb Rapid Test (colloidal gold)	CE-IVD	15	Intended for professional use only	Plasma	Alpha – unknown	96.19%	95.00%
				Serum	Beta – unknown		
				Venous blood	Gamma – unknown		
SARS-CoV-2 nAb Test Kit (colloidal gold)	CE-IVD	15	Intended for professional use only	Plasma	Alpha – no expected impact (*in silico* analyses)	90.70%	98.63%
				Serum	Beta – no expected impact (*in silico* analyses)		
				Venous blood	Gamma – unknown		
COVID-19 nAb Rapid Test Kit (whole blood/serum/plasma)	CE-IVD	15	Intended for professional use only	Plasma	Alpha – no expected impact (*in silico* analyses)	99.29%	99.76%
				Serum	Beta – no expected impact (*in silico* analyses)		
				Venous blood	Gamma – unknown		
2019-nCoV nAb Test Cassette (whole blood/serum/plasma)	CE-IVD	15	Intended for professional use only	Plasma	Alpha – no expected impact (*in silico* analyses)	94.17%	98.18%
				Serum	Beta – no expected impact (*in silico* analyses)		
				Venous blood	Gamma – unknown		
PRIMACOVID™ Covid-19 nAb Rapid Test	CE-IVD	10	Intended for self-testing (in development)	Plasma	Alpha – no expected impact (*in silico* analyses)	92.60%	97.40%
				Serum	Beta – unknown		
				Whole blood	Gamma – unknown		
SARS-CoV-2 nAbs Test Kit (dry fluorescence Immunoassay)	CE-IVD	15	Intended for professional use only	Plasma	Alpha – unknown	95.00%	98.00%
				Serum	Beta – unknown		
				Venous blood	Gamma – unknown		
SARS-CoV-2 nAb Rapid Test (fluorescence dry quantitative immunoassay)	CE-IVD	10	Intended for professional use only	Plasma	Alpha – no expected impact (*in silico* analyses)	97.40%	100.00%
				Serum	Beta – no expected impact (*in silico* analyses)		
				Venous blood	Gamma – no expected impact (*in silico* analyses)		
SARS-CoV-2 nAb Rapid Test (colloidal gold)	CE-IVD	15	Intended for professional use only	Plasma	Alpha – no expected impact (*in silico* analyses)	94.70%	100.00%
				Serum	Beta – no expected impact (*in silico* analyses)		
				Venous blood	Gamma – no expected impact (*in silico* analyses)		
Corona Virus (COVID-19) Combined (IgM/IgG/nAb) Rapid Test	CE-IVD	15	Intended for professional use only	Plasma	Alpha – unknown	99.00%	97.00%
				Serum	Beta – unknown		
				Venous blood	Gamma – unknown		
COVID-19 nAb Test Kit	CE-IVD	15	Intended for professional use only	Plasma	Alpha – no expected impact (*in silico* analyses)	95.40%	98.40%
				Serum	Beta – no expected impact (*in silico* analyses)		
				Venous blood	Gamma – unknow		
Wesail COVID-19 nAb Test Kit	CE-IVD	15	Intended for professional use only	Plasma	Alpha – no expected impact (*in silico* analyses)	95.40%	98.40%
				Serum	Beta – no expected impact (*in silico* analyses)		
				Venous blood	Gamma – unknown		
SARS-CoV-2 nAb Test Kit (colloidal gold)	CE-IVD	15	Intended for self-testing (version available)	Plasma	Alpha – no expected impact (*in silico* analyses)	98.13%	99.26%
				Serum	Beta – no expected impact (*in silico* analyses)		
				Whole blood	Gamma – unknown		
SARS-CoV-2 nAb Rapid Test (colloidal gold)	CE-IVD	10	Intended for professional use only	Plasma	Alpha – unknown	98.56%	99.65%
				Serum	Beta – unknown		
				Whole blood	Gamma – unknown		

nAb-LFA can be performed similar to other neutralization tests by diluting the sample multiple times in triplicate to calculate the inhibitory concentration 50 (IC_50_), effective concentration 50 (EC_50_) or PRNT_50_. However, for nAb-LFA, which is intended for individual home users, it is impossible to prepare blood samples at home at different dilutions accurately. Therefore, a single dilution must be selected that will cover the protective titer of nAb. Currently, the FDA recommends an nAb titer at least 1 : 160; however, the titer of 1 : 80 may be acceptable if no alternative matching units are available. For nAb-LFA, the optimal cut-off value should be the IC_50_ of the selected single dilution. Zhang *et al.* tested 80 COVID-19 plasma samples using nAb-LFA with an artificially set 50% inhibition at 1 : 12 dilution as the cut-off value and found that the final result was similar to the ELISA-based neutralization assay (*R*^2^ = 0.79).^321^ Wang *et al.* speculated that the nAb-LFA using a single dilution of the test sample could be designed to cover the range of color changes equivalent to 1 : 20 to 1 : 100 in PRNT.^322^ However, the cut-off value should be determined by large-scale clinical trials, such as the IC_50_ diluted sera for 1 : 10 for Japanese Encephalitis^323^ and 1 : 22 for Mumps.^324^

Overall, nAb-LFA is inexpensive and highly portable, which can be used to assist in the detection and longitudinal evaluation of nAbs in resource-poor areas where traditional virus neutralization tests or ELISA-based neutralization assays are not available. Considering that the design and manufacture of nAb-LFA are comparatively easy, even if the virus mutates, this can be addressed by modifying the labeled recombinant protein. However, the significant limitation of nAb-LFA is that it cannot facilitate the detection of nAb without high affinity for the S1 protein and a large number of nAbs with low affinity.

## 5. SARS-CoV-2 antigen detection

Since the beginning of the COVID-19 pandemic, nucleic acid amplification assays (*e.g.*, RT-PCR assays) have dominated to detect the SARS-CoV-2 virus. Thus far, clinical laboratories worldwide have performed over 3 billion molecular diagnostic tests for SARS-CoV-2. More than 850 million tests were performed in the United States (an average of 2.5 tests per person),^305^ although China has not yet reported complete testing data, but the absolute number should be greater than any other countries. The execution of mass nucleic acid amplification assays is technically challenging, labor-intensive, and dependent on efficient sample transport and reporting systems, all of which contribute to detection bias. Thus, to facilitate the diagnosis and treatment of COVID-19, antigen rapid diagnostic tests (Ag-RDTs) for SARS-CoV-2 have evolved rapidly, with at least 600 antigen testing kits now available worldwide and widely used in healthcare settings as well as high- and low-resource settings.^306^ An analysis on the performance of several mainstream test kits currently available in the market is shown in Table 8.

**Table 8:** Analysis on the performance of several mainstream SARS-CoV-2 antigen test kits

Method	Developer	Test	Sensitivity	Specificity	Antigen	Time (min)	Specimen
LFIA	SD Biosensor, Inc.	COVID-19 At-Home Test	95.3%	100.0%	SARS-CoV-2 NP	15–30	Human nasal sample
	InBios International Inc.	SCoV-2 Ag Detect Rapid Self-Test	85.7%	100.0%	SARS-CoV-2 NP	∼20	Human nasal sample
	Oceanit Foundry LLC	ASSURE-100 Rapid COVID-19 Test	89.00%	100.0%	SARS-CoV-2 NP	∼20	Human nasal sample
	PHASE Scientific International, Ltd	INDICAID COVID-19 Rapid Antigen Test	84.4%	96.3%	SARS-CoV-2 NP	∼20	Human nasal sample
	Nano-Ditech Corp.	Nano-Check COVID-19 Antigen Test	90.32%	100.0%	SARS-CoV-2 NP	15–20	Human nasal sample
	Siemens Healthineers	CLINITEST Rapid COVID-19 Antigen Self-Test	86.5%	99.30%	SARS-CoV-2 NP	∼15	Human nasal sample
	Abbott Diagnostics Scarborough, Inc.	BinaxNOW COVID-19 Ag Card Home Test	91.7%	100.00%	SARS-CoV-2 NP	15–30	Human nasal sample
	iHealth Labs, Inc.	iHealth COVID-19 Antigen Rapid Test Pro	88.2%	100.00%	SARS-CoV-2 NP	∼15	Human nasal sample
	iHealth Labs, Inc.	iHealth COVID-19 Antigen Rapid Test	94.3%	98.10%	SARS-CoV-2 NP	∼15	Human nasal sample
	Celltrion USA, Inc.	Celltrion DiaTrust COVID-19 Ag Home Test	86.7%	99.80%	SARS-CoV-2 NP and S-RBD antigens	∼15	Human nasal sample
	Xtrava Health	SPERA COVID-19 Ag Test	91.8%	96.90%	SARS-CoV-2 NP	∼15	Human nasal sample
	ANP Technologies, Inc.	NIDS COVID-19 Antigen Rapid Test Kit	95.1%	97.00%	SARS-CoV-2 NP	∼15	Human nasal sample
CLIA	Siemens Healthcare Diagnostics, Inc.	ADVIA Centaur SARS-CoV-2 Antigen (CoV2Ag)	85.1%	100.0%	SARS-CoV-2 NP	10	Human nasal sample
	Siemens Healthcare Diagnostics, Inc.	Atellica IM SARS-CoV-2 Antigen (CoV2Ag)	85.1%	100.0%	SARS-CoV-2 NP	26	Human nasal sample
	LIAISON SARS-CoV-2 Ag	DiaSorin, Inc.	97.00%	100%	SARS-CoV-2 NP	∼30	Human nasal sample

Antigen-based diagnostic tests are based on the principle of detecting viral proteins by antigen capture methods (*e.g.*, using antibodies and aptamers), and in clinical testing, are mainly used to detect protein fragments on or within the virus from NP swabs or nasal swabs. To date, most antigen tests use portable devices (*e.g.*, LFIA). Compared to RT-PCR, this type of test can detect active infection within 15 min, and it minimizes the risk of virus transmission by avoiding crowd gathering. Therefore, this method offers significant benefits in preventing early infection transmission. Besides, enzyme immunoassay techniques such as ELISA or CLIA on semi-automated or automated instruments also enable the detection of antigens with high-throughput. To date, many other antigen detection technologies have been developed for SARS-CoV-2 such as biosensors using nanotechnology, field-effect transistors (FETs), microfluidic platforms and electrochemical methods.

Given the current widespread use of Ag-RDT in clinical, community and home settings, the progress of antigen detection is summarized and its clinical application together with existing challenges is evaluated in this section.

### 5.1 SARS-CoV-2 NP detection

The most common protein in the structure of SARS-CoV-2 is NP, which is an evolutionarily conserved, highly immunogenic protein. NP is essential for the early replication of the virus in the host cell, which plays a major role in packaging viral RNA into a ribonucleoprotein (RNP) complex called nucleocapsid.^325,326^ When the virus enters the host cell, NP supports the replication of the viral RNA and releases the virus particles into the host cell.^327,328^ However, because NP is highly conserved among all coronaviruses, the specificity of its detection is low.^329^ NP is released in large quantities in the serum, nasopharyngeal aspirate, throat wash samples, feces and urine in the early stages of infection.^330^ Therefore, the detection of NP antigen may be an effective strategy for the early screening of patients with suspected SARS-CoV-2 infection.^331^

**Fig. 12:**
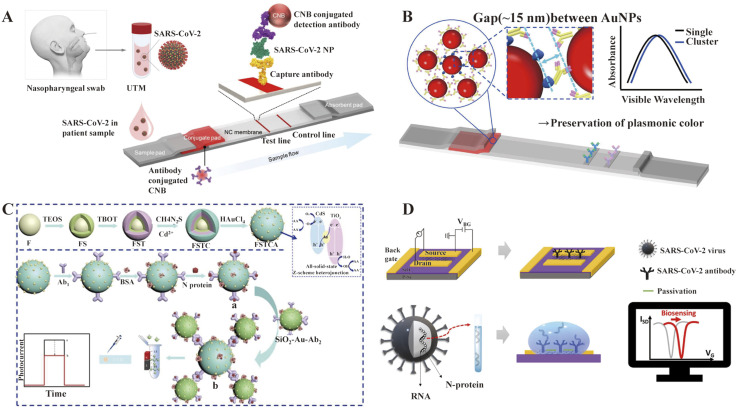
Schematic diagram of some strategies for SARS-CoV-2 NP assay. (A) Schematic illustration of the development processes of SARS-CoV-2-specific scFv-Fc fusion proteins based on phage display technology and LFIA-based biosensor using scFv-Fc fusion proteins. Reproduced with permission from *Biosens. Bioelectron.*, 2021, **175**, 112868, 10.1016/j.bios.2020.112868.^337^ (B) Structure of AuNP-based clusters LFIA. Reproduced with permission from *Biosens. Bioelectron.*, 2022, **205**, 114094, 10.1016/j.bios.2022.114094.^340^ (C) Preparation and detection process of signal-off photoelectrochemical immunosensor based on magnetic FSTCA. Reproduced with permission from *Sens. Actuators, B*, 2023, **374**, 132800, 10.1016/j.snb.2022.132800.^342^ (D) The Schematic of the device configuration and biosensing mechanism of the BN-GO gel FET NP biosensor. Reproduced with permission from *Biosens. Bioelectron.*, 2022, **210**, 114331, 10.1016/10.1016/j.bios.2022.114331.^352^

Currently, most Ag-RDTs are quantitative immunoassays that use SARS-CoV-2-specific antibodies to bind viral proteins and generate visual or fluorescent signals. This strategy is primarily performed in the LFA on a nitrocellulose membrane and provides assay results within 10 to 30 min.^332–335^ In LFIA, the probe that can specifically bind to NP is the key factor in determining the assay performance. To obtain high-performance immunoprobes, Yamaoka *et al.* used a wheat germ cell-free protein production system yielding a monoclonal antibody (mAb) that specifically binds to the SARS-CoV-2 NP.^336^ The modified antibodies not only had no cross-reactivity with other coronaviruses, but this mAb-based LFIA could also be used to detect variants 501Y.V1-V3. Kim's team also obtained four SARS-CoV-2 NP-specific single-chain variable fragment-crystallizable fragment (scFv-Fc) fusion antibodies by phage display.^337^ They screened the specific scFv-Fc antibody pair as the LFIA detection probe, and the developed cellulose nanobead LFIA platform could detect 2 ng of antigenic protein and 2.5 × 10^4^ pfu of cultured virus (Fig. 12A). In addition to probes, the color-labeling of LFIA can greatly affect the detection performance.^338,339^ Oh *et al.* synthesized a plasmonic exciton color-preserving (PLASCOP) gold nanocluster to detect NPs with an LOD 5.9 to 23.8-times lower than that of GNPs (Fig. 12B).^340^ Wang *et al.* developed a triple quantum dot shell (MagTQD) nanotag and integrated it in the LFIA system, enabling the simultaneous detection of the SARS-CoV-2 SP and NP antigens on one strip. The LOD for the two antigens in direct and enrichment modes was 1 and 0.5 pg mL^−1^, respectively.^341^ Novel nanomaterials can be used in the LFIA platform to design other detection formats. Guo *et al.* synthesized a versatile magnetic all-solid Z-scheme heterojunction (Fe_3_ O_4_@SiO_2_@TiO_2_@CdS/Au, FSTCA) nanocomposite, which has various advantages such as simplified separation and washing process, to improve the reproducibility and stability. Then, the authors developed a photoelectrochemical immunosensor to detect NP with a wide linear range of 10 pg mL^−1^ to 100 ng mL^−1^ and low detection limit down to 2.9 pg mL^−1^.^342^ The Si-FITC NPs prepared by Mao *et al.* not only solved the problem of reduced quantum yield during the coupling reaction but also achieved the sensitive detection of NP (LOD of 3 pg mL^−1^) (Fig. 12C).^343^ Besides antibody probes, aptamer probes were used in the NP assay.^344^ Zhang *et al.* constructed a sensor using an aptamer capable of detecting pM-level NP.^345^ In addition, other sensors are also being actively expanded for NP detection, such as FET sensors,^346,347^ microfluidic platforms^348,349^ and electrochemical sensors.^350,351^ Recently, Novodchuk *et al.* developed an FET sensor using boron/nitrogen co-doped graphene oxide gels (BN-GO gels), which could detect viral proteins with an LOD of 10 ag mL^−1^ (Fig. 12D).^352^

### 5.2 SARS-CoV-2 SP detection

SP is a glycoprotein (∼140 kDa) that consists of two subunits (S1 and S2) and forms a trimer on the viral membrane.^353^ Compared with other SARS-related coronaviruses, the nucleotide sequence similarity of the S gene is less than 75%.^71^ Therefore, SP may be one of the most valuable antigenic biomarkers for diagnosing COVID-19.

**Fig. 13:**
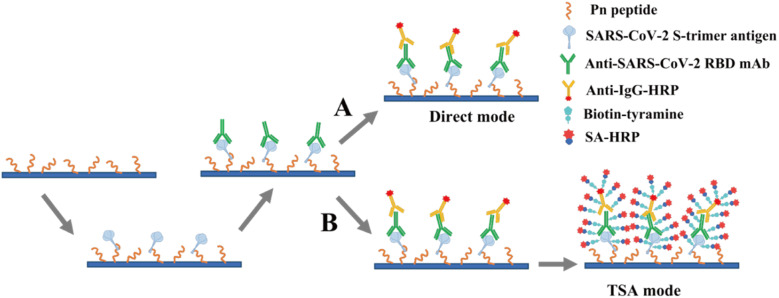
Schematic illustrating the p-ELISA to detect SARS-CoV-2 S antigen. (A) In the direct mode, the peptide Pn was used as the capture probe and an antibody (anti-SARS-CoV-2 RBD mAb) bound to the S-RBD was selected as the detection antibody, by which a “peptide-antigen-antibody” sandwich p-ELISA was constructed to detect SARS-CoV-2 SP antigen. (B) TSA mode. Upon catalysis by HRP, biotinylated tyramine is deposited at the site of signal amplification. Subsequently, SA-HRP is added to introduce more HRP in the positive microplate wells where the sample is tested, taking advantage of the high affinity between streptavidin and biotin. Reproduced with permission from *Sens. Actuators, B*, 2023, **387**, 133746, 10.1016/j.snb.2023.133746.^372^

Similar to the NP assay, most LFIA assays for SP are based on the hybridization of antigen–antibody immunoreactivity.^332,354–356^ However, Baker *et al.* found that N-acetylneuraminic acid had affinity and specificity for SARS-COV-2 SP, based on which they developed a paper-based LFA and verified its feasibility using pseudovirus.^357^ Similarly, Kim *et al.* found that glycoproteins also can bind to the SARS-COV-2 S protein, based on which they developed an SP test platform called GlycoGrip.^358^ These studies broaden the idea of exploring new biological probes. More recently, several nanoscale integrated architectures based on optical and electronic systems have been introduced to detect SP with much higher sensitivity, such as terahertz plasmonic biosensor,^359^ gated graphene-enhanced FET-based biosensors,^360^ In_2_O_3_ nanoribbon transistors biosensor,^361^ and electrical-double-layer gated FET-based biosensor.^347^ Generally, most of these devices have favorable sensitivity and possess the advantages of low cost, simplicity, easy miniaturization and mass fabrication. However, there is still a continuous demand for fast, ultrasensitive biosensors to detect SP. SERS is known as an ultrasensitive molecular spectroscopy technique that is not interfered by water, giving it a distinct advantage in identifying biological samples.^362,363^ Compared to LFIA, photothermal methods and electrical biosensors, SERS-based immunoassay techniques do not require sample pretreatment and are highly sensitive for the detection of trace bioparticles.^363,364^ Currently, SERS technology has been well developed and applied in SP detection.^365–367^ Besides direct testing for unprocessed samples,^368^ SERS platforms combined with other techniques have also been developed. Huang *et al.* developed a deep learning-based SERS technique for the on-site detection of SP from human throat swabs and sputum with an accuracy of 87.7% in 20 min.^369^ Yang *et al.* introduced machine algorithms in SERS technology to further develop a sensor capable of detecting SARS-CoV-2 SP and differentiating among 13 respiratory viruses with >99% accuracy.^370^ Shim *et al.* proposed a sensing platform based on core–shell SERS with single nanoparticle (SNP)-based digital SERS analysis capability. This platform has a better detection range and lower detection limit than conventional ELISA, which can distinguish multiple mutants.^371^ Peptides have merits over antibodies including smaller size, stability and scaled-up preparation. Recently, our group screened a peptide probe Pn that can specifically recognize SP through phage display. By combining a specific peptide with the tyramine signal amplification (TSA) method, an ultra-sensitive peptide-ELISA (p-ELISA) detection method was developed, capable of detecting SARS-CoV-2 SP as low as 0.4 pg mL^−1^ and SARS-CoV-2 pseudoviruses with 3 TCID _50_ per mL (Fig. 13).^372^

#### 5.2.1 SARS-CoV-2 S1 protein detection

**Fig. 14:**
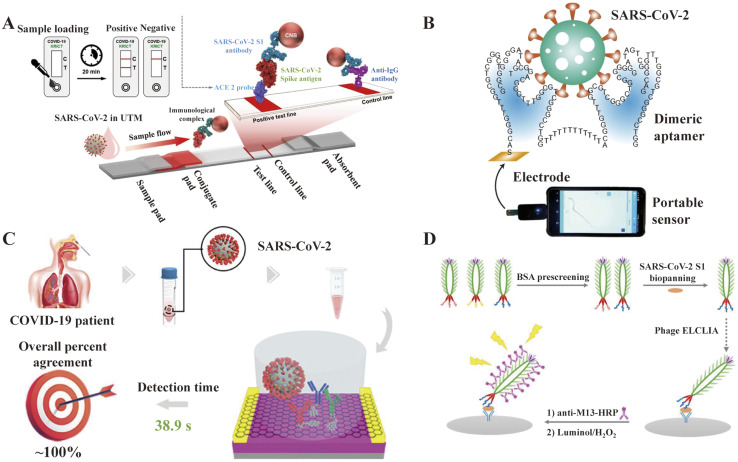
Schematic diagram of some strategies for S1 protein detection. (A) Schematic of an ACE2-based LFIA consisting of a sample pad, conjugate pad, nitrocellulose membrane, and absorbent pad. The test line placed on the nitrocellulose membrane contains ACE2 to detect SARS-CoV-2 SP. Reproduced with permission from *Biosens. Bioelectron.*, 2021, **171**, 112715, 10.1016/j.bios.2020.112715.^374^ (B) Platform for the rapid electrochemical detection of S1 protein with high-affinity dimer aptamer. Reproduced with permission from *Angew. Chem.*, 2021, **60**, 24266–24274, 10.1002/anie.202110819.^388^ (C) Multiantibody FET sensor. Reproduced with permission from *J. Am. Chem. Soc.*, 2021, **143**, 19794–19801, 10.1021/jacs.1c08598.^386^ (D) Schematic diagram of antibody/S1/specific peptide-displayed phage/HRP-M13 antibody-based ELCLIA. Reproduced with permission from *Anal. Chem.*, 2022, **94**, 11591–11599, 10.1021/acs.analchem.2c01988.^387^

The N and S proteins of SARS-CoV-2 have very low cross-reactivity between epidemic coronaviruses and common human coronaviruses, but studies have shown that the S1 structural domain of the S protein has much lower cross-reactivity. Thus, S1 is considered to be more specific than the natural homologous trimer of the S protein.^373^ Because of the strong binding ability of ACE2 and S1 protein, Lee *et al.* developed an LFIA platform for detecting SARS-CoV-2 S1 by pairing ACE2 and S1-mAb (Fig. 14A). This platform demonstrated the ability to detect SARS-CoV-2 S1 and is comparable to antibody sandwich methods (LOD of 1.86 × 10^5^ copies per mL).^374^ Subsequently, they also developed a dual-mode multifunctional LFIA platform using the combination of ACE2 and S1-mAb. In the “binding mode”, the platform could distinguish wild-type SARS-CoV-2 S1 from Alpha and Beta variants by color differences. In “blocking mode”, it could detect nAbs in COVID-19 patients.^375^ The readout mode can also be more versatile in addition to the pluripotency of the detection mode. By mixing 20 nm AuNPs and quantum dots (QDs) on SiO_2_ to form a monolayer shell coating, Han *et al.* prepared a bifunctional immunolabel with both strong colorimetric and fluorescent signals. The as-prepared LFIA for detecting the S1 protein has dual-mode of colorimetric and fluorescent reading.^376^ For S1 protein detection, aptamer probes were also used as biorecognition elements.^377^ Li *et al.* obtained two aptamers with pM affinity for SARS-CoV-2 S1 by *in vitro* selection assay, by which a colorimetric sandwich sensor was constructed to detect 400 fM S1 protein in saliva samples.^378^ Some electrochemical platforms has also been established for S1 protein detection due to its good sensitivity, speed, low cost and stability.^379,380^ Zhang *et al.* constructed a bivalent aptamer probe by linking two aptamers, by which a CoV-eChip electrochemical platform was developed with an LOD of 1000 copies per mL S1 protein, which had clinical sensitivity of 80.5% and specificity of 100% (Fig. 14B).^381^ Other methods were explored to detect S1 proteins.^382–385^ Dai *et al.* developed a multi-antibody transistor assay for sensitive and highly accurate antigen pool testing.^386^ The multi-antibodies captured the SARS-CoV-2 spike S1 proteins with different configurations, resulting in an antigen-binding affinity as low as 0.34 fM. The LOD reached 3.5 × 10^−17^ g mL^−1^ S1 protein in artificial saliva, which is 4–5 orders of magnitude lower than existing transistor sensors (Fig. 14C).^386^ Our group biopanned a peptide specific to SARS-CoV-2 S1 by pIII phage display. Then, an enzyme-linked CLIA (ELCLIA) was developed by using this peptide-displayed phage as a bifunctional probe capable of SARS-CoV-2 S1 recognition and signal amplification, which could detect 78 pg mL^−1^ of SARS-CoV-2 S1 and 60 transduction units (TU) per mL of SARS-CoV-2 pseudoviruses (Fig. 14D).^387^

Functionally, the binding of RBD to ACE2 is crucial for the SARS-CoV-2 virus to enter human cells. SARS-CoV-2 S-RBD shares only 70% sequence identity with SARS-CoV S-RBD, and the latter has been evaluated for vaccine and therapeutic drug development.^389^ Therefore, the S-RBD of SARS-CoV-2 is an excellent target for diagnosis and therapeutic intervention. Many of the technologies described above were developed and designed to detect RBD to diagnose SARS-CoV-2. For example, Raman spectroscopy,^390–393^ electrochemical methods,^394–396^ localized surface plasmon resonance (LSPR)^397^ and lateral flow tomography.^333,398^ Recently, Yang *et al.* reported a high-affinity SARS-CoV-2 Omicron variant RBD-binding aptamer (SCORe) that binds to the Omicron BA.1 and BA.2 variant RBD with affinity at the nM level. Multiplex flow assays were developed to distinguish the Omicron variant from wild-type down to 100 pM.^399^ In addition, Ravalin *et al.* developed a biosensor that uses binding-activated tandem split-enzyme technology to detect RBD. The biosensor is a single-component, recombinant, luminescent sensor that can be expressed in laboratory strains of *Escherichia coli* and *Saccharomyces cerevisiae*, which was detected by a regular cell phone camera to record the chemiluminescent signal.^400^ Murtaza *et al.* obtained a new high-affinity aptamer for RBD by computer methods to fabricate a photonic crystal (PC)-decorated aptamer sensor, for which the aptamer is cross-linked in the polyacrylamide hydrogel network and selectively binds to SARS-CoV-2 in saliva samples. The binding reaction can be visually monitored by swelling of the hydrogel and color generated by light diffraction from the PC and can be quantified by the diffraction ring diameter or spectroscopy.^401^

Several studies suggest that in some coronaviruses, NTD may recognize specific glycosyl groups during initial attachment and play an essential role in the transition of S proteins from pre-fusion to post-fusion. Thus, it may be involved in the regulation of viral infection.^402–404^ Currently, most variants are mutated mainly in the RBD region.^405^ Therefore, the NTD assay may diagnose multiple variants more effectively.

Kacherovsky *et al.* obtained the SNAP1 aptamer with good affinity to NTD, which was applied in LFIA and ELISA to detect SARS-CoV-2 down to 5 × 10^5^ copies per mL.^406^ Recently, this team obtained another aptamer SNAP4 binding NTD with 2-fold higher affinity than that of SNAP1. In addition, the constructed sandwich LFIA with both SNAP1 and SNAP4 could detect 10^6^ copies per mL SARS-CoV-2.^407^

The analytical performance of some antigen detection methods are summarized in Table 9. Currently, many antigen detection kits based on these technologies have been approved for clinical testing to tackle SARS-CoV-2. The WHO suggests that all countries to use high-quality rapid antigen tests to effectively respond to the global COVID-19 pandemic.^408^

**Table 9:** Analytical performance of some SARS-CoV-2 antigen assays

Methods	Target antigen	Sample	Linear range	LOD	Detection time	Ref
LFIA	NP	Clinical isolate of SARS-CoV-2	—	2.5 × 10^4^ pfu	20 min	337
Double-antibody sandwich ELISA	NP	Recombinant protein	—	0.78 ng mL^−1^	About 1 day	409
Nanomechanical sensor	S1, NP	Nasopharyngeal swab	—	0.71 ng mL^−1^	≤5 min	410
Microfluidic magneto immunosensor	NP	Whole serum	1–10 000 pg mL^−1^	0.23 ng mL^−1^	<1 h	16
Half-strip LFA	NP	Recombinant protein	—	0.65 ng mL^−1^	About 10 min	411
LFIA	NP	Human serum or urine	—	1.0 ng mL^−1^	<15 min	345
MagPlex fluid array assays	NP	Recombinant protein	—	0.05 ng mL^−1^	<1 h	412
Cotton-tipped electrochemical immunosensor	NP	Clinical samples	1–1000 ng mL^−1^	0.8 pg mL^−1^	About 25 min	350
Electrochemical biosensor	NP	Recombinant protein	1 pg mL^−1^–100 ng mL^−1^	0.4 pg mL^−1^	About 20 min	413
Bright fluorescent probe assay	NP	Nasopharyngeal swab samples	—	30 ng mL^−1^	—	414
Electrochemical sensor	NP	Clinical samples	2.22–111 fM	15 fM	About 20 min	415
Chemiluminescent functionalized magnetic nanomaterial	NP	Recombinant protein	0.1 pg mL^−1^–10 ng mL^−1^	69 fg mL^−1^	—	416
Immunochromatographic assay augmented with silver amplification	NP	Nasopharyngeal swab	—	6.3 ×10^4^ copies per mL	About 15 min	336
Nanozyme-linked immunochromatographic sensor	NP	Clinical samples	0.05–1.6 ng mL^−1^	0.026 ng mL^−1^	≤25 min	417
Smartphone-based nanozyme-linked immunosorbent assay	NP	Serum samples	30 pg mL^−1^–3 ng mL^−1^	10 pg mL^−1^	<1 h	418
AuNP-based plasmonic sensor	NP	Recombinant protein	150–550 ng mL^−1^	150 ng mL^−1^	<5 min	419
Colorimetric LFIA	NP	Human saliva	0–1000 ng mL^−1^	54 TCID_50_ per mL	15 min	340
MagTQD-LFIA	SP, NP	Saliva and nasal swab samples	1 pg mL^−1^–1000 ng mL^−1^	0.5 pg mL^−1^	10 min	341
Photoelectrochemical immunosensor	NP	Recombinant protein	10 pg mL^−1^–100 ng mL^−1^	2.9 pg mL^−1^	—	342
Ratiometric fluorescent Si-FITC nanoprobe for immunoassay	NP	Human serum	0.02–50 ng mL^−1^	0.003 ng mL^−1^	—	343
Electrical-double-layer gated FET-based biosensing	NP	Artificial saliva	0.4 ng mL^−1^–400 ng mL^−1^	0.14 ng mL^−1^ (2.96 pM)	65 min	347
FET	NP	Calibration samples	16 fg mL^−1^–16 pg mL^−1^	0.016 fg mL^−1^	5 min	346
Handheld microfluidic filtration platform	NP	Nasal swab samples	1 × 10^3^–1 × 10^6^ particles per mL	100 copies per mL	15–30 min	348
Microelectrode array chip	NP	Throat swabs	10^−5^–10^−2^ ng mL^−1^	3.16 × 10 ^−6^ ng mL^−1^	15 s	349
Electrochemical immuno-biosensing	NP	Spiked sample	1–10 000 pg mL^−1^	21 fg mL^−1^	15 min	351
Paper-based electrochemical platform	NP	Serum sample	1–100 ng mL^−1^	0.11 ng mL^−1^	—	420
FET biosensor	SP	Cultured SARS-CoV-2	—	1.6 × 101 pfu mL^−1^	—	360
Microfluidic biochip	SP	Serum	—	0.3 pg mL^−1^	40 min	421
Electrochemical detection	SP	Recombinant protein	1 pg mL^−1^–10 ng mL^−1^	1 pg mL^−1^	About 10 min	422
Electrochemical biosensor	SP	Saliva, artificial nasal, UTM	0.25 fg mL^−1^–1 μg per mL	0.04 fg mL^−1^	20 min	423
G-quadruplex aptamer-based sensor	SP	Nasopharyngeal swab specimens	0.5–125 nM	2 nM	—	424
Graphite electrode functionalized with AuNPs	SP	Recombinant protein	1 pg mL^−1^–1 ng mL^−1^	229 fg mL^−1^	6.5 min	425
WSe2 based-FET	SP	Recombinant protein	25 fg μL^−1^–10 ng μL^−1^	25 fg μL^−1^	—	426
SERS (Au/Si with labels)	SP	Untreated saliva	1 fg mL^−1^–1ng mL^−1^	0.77 fg mL^−1^	About 5 h	427
Multi-antibody transistor assay	S1	Saliva	—	3.5 × 10^−17^ g per mL	38.9 s	386
Nanozyme chemiluminescence paper test	SP	Nasopharyngeal swab sample	0.1–100 ng mL^−1^	0.1 ng mL^−1^	30 min	428
Lateral flow device	SP	Pseudotyped lentivirus	—	1.5 × 10^4^ TU per mL	<30 min	357
Cell surface-inspired universal sensor	SP	Recombinant protein	3.13–50 μg mL^−1^	3.13 μg mL^−1^	< 30 min	358
Functionalized terahertz plasmonic metasensor	SP	Recombinant protein	4–12 fM	∼4.2 fM	—	359
In_2_O_3_ nanoribbon transistor	SP	Recombinant protein in UTM	—	100 fg mL^−1^	—	361
Single-particle SERS immunoassay	SP	Saliva	10^5^–10^7^ genomes per mL	1.94 × 10^3^ genomes per mL or 4.7 fg per mL	—	365
SERS	S1	Recombinant protein	0.01 ng mL^−1^–100 ng mL^−1^	10 pg mL^−1^	2 h	366
Plasmonic nanostructure-enhanced Raman scattering	SP	Recombinant protein	1.0 pg mL^−1^–1.0 mg mL^−1^	1 fg mL^−1^	—	367
SERS-based biosensor	SP	Saliva	10 fg mL^−1^–10 ng mL^−1^	6.07 fg mL^−1^	—	368
Deep learning-based SERS	SP	Throat swabs or sputum	—	10 fg mL^−1^	<20 min	369
SERS	SP	Saliva	400 PFU mL^−1^–10^5^ PFU mL^−1^	391 PFU mL^−1^	—	370
Bumpy core–shell SERS	SP	Recombinant protein and clinical sample	0.1 fg mL^−1^ to 10 ng mL^−1^	0.1 fg mL^−1^	< 30 min	371
Peptide-ELISA	SP	Recombinant protein and pseudoviruses	—	1.4 fM, 3TCID_50_ per mL	2 days	372
Fluorescence LFIA	SP and NP	Saliva and nasal swab	1 pg mL^−1^–100 ng mL^−1^	0.5 pg mL^−1^	10 min	374
LFIA	S1	Nasal swab	1.86 × 10^5^ copies per mL	—	20 min	375
LFIA	S1	Nasopharyngeal swabs	1 × 10^6^ pfu mL^−1^–1 × 10^5^ pfu mL^−1^	500 pg mL^−1^	20 min	376
Colorimetric and fluorescent dual-functional LFIA	S1	Saliva samples	33 pg mL^−1^–1 ng mL^−1^	37 pg mL^−1^	30 min	378
Impedimetric sensor	S1	—	0.0059 fg mL^−1^–0.020 fg mL^−1^	7.2 fg mL^−1^	—	380
Electrochemical biosensor	S1	Saliva swab	0.5–5 ng mL^−1^	0.15 ng mL^−1^	—	388
Electrochemical biosensor	S1	Saliva swab	—	1000 copies per mL	10 min	382
SPR biosensor	S1	Artificial saliva and human serum samples	0.1 pg mL^−1^–1000 ng mL^−1^	12 fg mL^−1^	—	429
Electrochemical sensor	S1	Saliva swab	0.1 fg mL^−1^ to 5.0 pg mL^−1^	4.12 fg mL^−1^	2–3 min	383
Immunoassay technique	S1	Mid-turbinate swabs and exhaled breath aerosol samples	1 ag mL^−1^–1 μg mL^−1^	60 copies per mL	0.6 s	384
Functionalization of graphene oxide-glazed double-interdigitated capacitive biosensing platform	S1	Recombinant protein	1.0 mg mL^−1^–1.0 fg mL^−1^	1 fg mL^−1^	3 s	385
Phage-based enzyme-linked CLIA	S1	Saliva swab	—	60 TU mL^−1^	—	387
LFIA	S1	Nasal swabs	—	1.86 × 10^5^ copies per mL	About 10 min	430
Cell-based biosensor	S1	Recombinant protein	10 fg–1 μg mL^−1^	1 fg mL^−1^	3 min	431
Electrochemical biosensor	S1	Recombinant SARS-CoV-2	—	20 μg mL^−1^	∼45 min	432
AuNP-based immunochromatographic strip	S-RBD	Recombinant protein	62.5–4000 ng mL^−1^	62.5 ng mL^−1^	15–30 min	433
Electrochemical sensor	SP and NP	Untreated saliva	—	19 ng mL^−1^	About 2 h	434
CNT-FET	S1	Recombinant protein	0.1 fg mL^−1^–5.0 pg mL^−1^	4.12 fg mL^−1^	2–3 min	429
Electrochemical sensor	S1	Nasopharyngeal samples	—	64 fM	15 min	435
Impedimetric biosensor	S-RBD	Nasal secretions	0.0012–120 pg mL^−1^	0.58 fg mL^−1^	About 2 h	436
ACE2-SWCNT nanosensors	S-RBD	Virus-like particles	—	12.6 nM	About 90 min	437
Nanozyme chemiluminescence paper	S-RBD	Pseudovirus	0.2–100 ng mL^−1^	0.1 ng mL^−1^	15 min	428
SERS	S-RBD	Saliva	—	1 nM	—	390
SERS	S-RBD	Recombinant protein	—	6.4 × 10^4^ molecules	—	391
SERS	S-RBD	Recombinant protein	—	1 fM	—	392
SERS	S-RBD	Recombinant protein	1.25 to 20 ng mL^−1^	0.625 ng mL^−1^	≤15 min	393
SERS	S-RBD	Recombinant protein	0.0012–120 pg mL^−1^	0.58 fg mL^−1^	—	394
Photonic crystals	S-RBD	Saliva swab	12.15 ng mL^−1^–13.25 ng mL^−1^	12.15 ng mL^−1^	5 min	401
Luminescent biosensor	S-RBD	Both recombinant form and cultured virus	0.1 nM and 10 nM	0.1 nM	—	400
Electrochemical immunosensor	S-RBD	Nasopharyngeal samples	1 fM–1 μM	0.73 fM	≤ 30 s	395
Lateral flow assays	S-NTD	UV-inactivated SARS-CoV-2 virus	—	5 × 10^5^ copies per mL	—	406
Aptamer sandwich lateral flow assay	S-NTD	Inactivated SARS-CoV-2 virus	—	10^6^ copies per mL	1 h	407

### 5.3 Clinical applications and challenges

In clinical testing, antigen detection is mainly used in the acute infection phase of SARS-CoV-2, *i.e.*, testing of samples within 5–7 days of the onset of symptoms in the suspected population (Omicron variant infection can be considered within 5 days). According to the WHO, the incubation period is an average of 3 days for the Omicron variant and 5–6 days for other SARS-CoV-2 variants.^438^ During incubation, virus shedding occurs in individuals who were infected with SARS-CoV-2. Clinical studies by Harvard Medical School showed that virus shedding was the highest in those infected with the Omicron variant within 3–6 days after onset.^439^ It is also revealed that the virus was undetected 10 days after onset in COVID-19 vaccine recipients infected with the Omicron variant. These studies suggest that antigen detection faces a limited window of optimal detection time, and therefore early infection and latency may be missed. However, according to viral culture research, SARS-CoV-2 may only replicate 10 to 14 days after the onset of symptoms. Antigen-based assays remain positive for 5 to 12 days after symptom onset and perform better in individuals with high viral loads.^440^ Thus, antigen-based assays correlate better with replication-competent SARS-CoV-2, which can provide information about potential transmissibility. Although hundreds of antigen-based tests are available for clinical testing worldwide, they are generally less sensitive than molecular tests compared to the reference standard of laboratory-based RT-PCR tests, especially in populations with low or no replication of viral load-capable viruses. Dinnes *et al.* summarized data from five studies involving 943 samples, with an average sensitivity of 56.2% (95% CI, 29.5–79.8%) and an average specificity of 99.5% (95% CI, 98.1–99.9%) for antigen detection compared to an average sensitivity of 95.2% (95% CI 86.7% to 98.3%) and an average specificity of 98.9% (95% CI, 97.3% to 99.5%) for molecular assays.^441^ After comparing Ag-RDT with viral culture and RT-PCR methods, Mak *et al.* concluded that the LOD of Ag-RDT was approximately 10^3^-fold lower than the culture-based SARS-CoV-2 assay and 10^5^-fold lower than the molecular assay.^442^ Mertens *et al.* stated that the sensitivity of Ag-RDT increased to 74.8% when samples with high viral loads (samples with RT-PCR CT values of ≤25) were evaluated, while the overall sensitivity was 57.6% when all the samples were considered.^443^ Due to the false negatives caused by antigen testing sensitivity, antigen testing is currently only used as an adjunct to RT-PCR testing. However, the potential application of antigen testing cannot be ignored. According to the recommendations of the WHO and CDC,^444^ anyone with symptoms of COVID-19 should be tested for SARS-CoV-2. Asymptomatic individuals in close contact with someone known or likely to be infected with SARS-CoV-2 should undergo diagnostic testing. In addition, asymptomatic individuals in a high-transmission risk setting should also be tested.

Presently, the global manufacturing, delivery, and implementation of antigen tests remain challenging. Firstly, antigen testing may not yield accuracy comparable to laboratory-based molecular testing, and thus diagnostic tests should be available to underserved and affordable populations. Secondly, although home-based Ag-RDTs have increased antigen testing, Ag-RDTs performed by trained healthcare providers have proven to be more accurate than that performed by untrained individuals. Therefore, test kit instructions should be carefully followed by those performing testing at home.

## 6. Tackling SARS-CoV-2 variants

### 6.1 Evolution of SARS-CoV-2

**Fig. 15:**
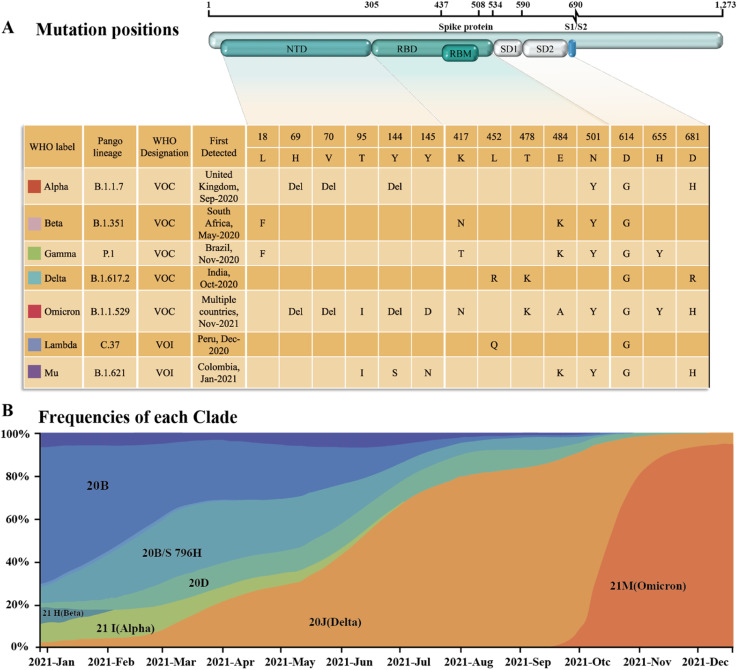
SARS-CoV-2 variants: constituent mutations and the frequencies of clade in each month of 2021. (A) Most frequent mutations in VOCs and VOIs. Spike protein residues are mapped to their associated domain within the spike protein, as shown in various color bars above the table. The first row of numbers in the table represents the mutated positions in the variants. The letters in the second row indicate the original amino acid sequence at that position. (B) Variation in the frequency of the different clades occurring worldwide over time. Among them, the frequency of Omicron has increased rapidly since its appearance.

New variants caused by viral mutations are emerging and spreading rapidly worldwide. Coronaviruses contain a special nucleic acid exonuclease (ExoN), which increases the replication fidelity of their genetic material by approximately 15-fold *in vitro*;^445^ however, when variants with different mutations infect the same host, recombination between them probably results in new SARS-CoV-2 variants. The accumulation of mutations will then gradually lead to SARS-CoV-2 diversity.^446^ In addition, human cytidine deaminases (APOBECs/ADARs) are also involved in the editing of SARS-CoV-2 transcriptome RNA, which also accelerates the SARS-CoV-2 diversity.^447^ The evolutionary rate of SARS-CoV-2 was estimated to accumulate 1–2 nucleotide mutations per month.^448^ Thus far, many variant strains have emerged, which contain constellations of mutations rather than a single site. Most mutations occur at similar locations and exhibit comparable properties, suggesting the recombination and evolution of SARS-CoV-2 in individuals. Although most mutations are harmless or neutral, some may result in increased disease severity, evasion of immune responses, reduced effectiveness of antiviral therapy, or enhanced ability of the virus to infect new animal hosts.^449^ Due to the growing emergence of variant strains, WHO designates variants that cause altered virus behavior as variants of interest (VOI) or VOC to prioritize global surveillance and research (Fig. 15A).^450^

Beginning in April 2020, the original SARS-CoV-2 strain was replaced by a variant called D614G (aspartate to glycine at position 614 of the S protein).^451^ By June 2020, the virus with the D614G mutation rapidly became the dominant variant worldwide, which was justified to be more infectious than the original strain by subsequent studies.^452^ Currently, both VOC and VOI have this mutation site. In October 2020, sequencing analysis in the UK detected an emerging variant, later called B.1.1.7 or 20I/501Y.V1. This variant contains ten mutations in the S protein.^453^ Among them, N501Y appeared to further increase the interaction of the S protein with ACE2, making this variant approximately 70% more transmissible than the original strain.^454,455^ Although the impact of this variant on disease severity remains uncertain, the B.1.1.7 variant may still lead to a dramatic increase in hospitalizations and deaths due to its high transmissibility. This was also confirmed in a retrospective observational study by Stirrup *et al.*^456^ The B.1.351 variant (20H/510Y.V2) was initially detected in South Africa in the end of 2020,^457^ while the P.1 variant (20J/501Y.V3) emerged in Brazil in November 2020.^458^ These variants possess three mutations (K417N/T, E484K, and N501Y) located on the RBD of the S protein, are associated with increased transmission potential. In addition, these two variants can mediate immune escape against protective antibodies produced by humoral immunity and vaccination.^459,460^ The Delta variant was first detected in India in December 2020, exhibiting a greater ability to evade the host's immune system than the original strain, which resulted in its rapid global spread and emergence as the dominant strain in numerous countries within just a few months after May 2021.^461^ There are multiple mutation sites in the S protein of the Delta variant (T19R, L452R, T478K, D614G, P681R and d960N and deletions at positions 157 and 158), with L452R and P681R being the two most notable mutations. The mutation at position 452 significantly increases the affinity of B.1.617.2 for ACE2, potentially allowing it to evade the vaccine-induced production of protective antibodies that bind to the S protein.^462^ Other studies showed that the mutation allows the B.1.617.2 variant to evade CD8 T cells. In addition, the mutation at position 681 helps cleave the precursor S protein into an activated form, permitting better fusion and integration of the virus into the host cell.^463^ The reasons for this may be that the B.1.617.2 variant has strong propagation and immune escape ability. The B.1.1.529 variant, later named Omicron, was first reported to the WHO on November 24, 2021, and quickly classified as a VOC just 2 days later. This variant contains an unusually high number of mutations, with over 30 identified in its S protein genes, including many located in the RBD and NTD regions.^464^ Among them, the deletion of 69/70 led to false negative results for S detection in TaqPath test.^465^ K417N and E484A enhanced the immune escape ability.^466^ G496S, Q498R, N501Y and Y505H significantly increased the binding affinity for ACE2,^464^ resulting in loss or reduction of neutralization, while H655Y, N679K and P681H enhanced their propagation properties.^467^ Currently, increasing evidence supports that this variant is more transmissible than B.1.617.2 and has surpassed its prevalence within one month of discovery (Fig. 15B).

Since the emergence of the Omicron variant, SARS-CoV-2 has continued to mutate. Currently, over 500 sub-lineages of the Omicron variant are in circulation, with the BA.1, BA.2, and BA.3 strains being the most common.^468^ The BA.1 strain has 37 mutations in its spike protein, while the BA.2 and BA.3 strains have 31 and 33 mutations, respectively. Among these mutations, 21 mutations are commonly shared by all three lineages, including N501Y and Q498R, to enhance the virus's binding affinity with the ACE2 receptor, and H655Y, N679K and P681H, which are thought to increase its transmission capability.^469^ Recently, the XBB.1.5 variant, recombining the BA.2.10.1 and BA.2.75 sub-variants, has been widely circulating in the United States.^470^ According to the WHO, XBB has been identified in at least 70 countries/regions, leading to a surge in infections in some parts of Asia, including India and Singapore.^471^ However, overall, the pathogenicity of the Omicron variant is not as severe as that of the Delta variant, which may be due to various factors such as the virus replicating more efficiently in the upper respiratory tract and the steady improvement of population immunity worldwide.

### 6.2 Sequencing for identification of SARS-CoV-2 variants

Genomics is the only method to identify and characterize new variants and clarify existing types. Whole genome sequencing (WGS) is an essential method for the genetic identification of viruses, allowing untargeted, unbiased sequencing of nucleic acids in a sample, and thus viral RNA or DNA can be identified if a sufficiently high copy number of DNA or RNA is present relative to other sources.^472,473^ The tiled amplicon method or shotgun sequencing is usually used in sequencing, which can also be compared with other circulating strains.^474^ Three groups used this method to identify the causative pathogen of COVID-19 at the beginning of the outbreak.^475^ With the emergence of various variants, this approach has also been employed to detect the variations and analyze the patterns and factors that influence the spread of these variants.^16,476^ The implementation of WGS can be found in WHO's Genomic sequencing of SARS-CoV-2: a guide to implementation for maximum impact on public health. However, although sequencing costs have decreased significantly, WGS is still relatively expensive compared to other molecular diagnostic techniques (*e.g.*, RT-PCR and isothermal amplification). In addition, WGS is a resource-intensive method that can generate results in several days. Therefore, it cannot be routinely used to diagnose various variants. Sanger-based or amplicon-based NGS can sequence all or part of the genes of a variant and is an effective alternative method for identifying and characterizing multiple variants. HiSpike, developed by Fass and colleagues, can identify variants within the S gene for the 20I/501Y.V1 and 20H/501Y.V2 variants with the help of a small Illumina MiSeq instrument.^477^ Compared with the conventional sequencing method, HiSpike improves the detection throughput and significantly reduces the detection costs and time. In another study, Castañeda developed ADSSpike similar to HiSpike. Compared to HiSpike, ADSSpike also sequences the S gene, but it uses deep sequencing (700 read depth) to identify SARS-CoV-2 mutants, capable of identifying variant-specific single nucleotide polymorphism (SNP) with a Ct value of up to 30.17 for the variant characterized.^478^ However, both strategies require tens of primer pairs to detect variants to cover the entire S gene. In fact, a dedicated SARS-CoV-2 variant diagnostic and assay monitoring strategy deployed globally will require cheap instruments and a reduced number of reagents to allow long-term storage. This problem was well addressed by Stüder *et al.*, who used long DNA fragments compatible with nanopore DNA sequencing technology.^479^ The solution requires only four primers targeting the Spike region, one random primer for the cDNA step, and a common Gibson sequence for real-time diagnosis and variant tracking on a portable MinION DNA sequencer. To further improve the detection throughput of sequencing, the pooling of samples has been attempted using primer barcodes. Cohen-Aharonov *et al.* used primers with barcodes to reverse transcribe and amplify RNA extracted from samples in one step, pooled and sequenced the resulting amplicons and identified infected individuals from all pooled test samples by subject-specific barcodes.^480^ The feasibility of this strategy for variant detection was verified by pooled testing of 960 samples. Chappleboim *et al.* also developed ApharSeq based on a pooled sample sequencing strategy, which combined samples as early as possible by hybridizing barcode primers.^481^ This allows multiplex reverse transcription, PCR and sequencing of hundreds of mixed samples to detect and classify variant sequences with high sensitivity and negligible contamination. Validation of the method on hundreds of clinical samples demonstrated excellent results (detection limit of Ct33, specificity > 99.5%) and a significant reduction in detection cost. The WGS and NGS methods for sequencing SARS-CoV-2 variants face the same challenges of expensive equipment and the need for specialized bioinformatics analysis, but these methods provide the basis for other detection technologies.

The SARS-CoV-2 genomic sequence data obtained by sequencing can help identify viral proteins that may be strongly antigenic and indicate how to generate these antigens for serological analysis. When SARS-CoV-2 acquires genomic substitutions, a spectrum with altered antigenic properties may emerge, indicating that serological assays cannot detect an infected individuals because the antigen used in the assay differs from the antigen the individual was exposed to. In addition, the rapid release of mutant sequences is important for designing primers and probes for molecular assays. These genomes are necessary for the design of primers and probes, and mismatches between primers or probes and corresponding binding sites within the SARS-CoV-2 genome may reduce the sensitivity of molecular diagnostic methods or lead to false negatives. As SARS-CoV-2 continues to acquire genetic changes, the continued generation and sharing of the viral genome are critical for monitoring the expected sensitivity of various diagnostic assays at different sites.

### 6.3 Other molecular assays for the detection of SARS-CoV-2 variants

**Fig. 16:**
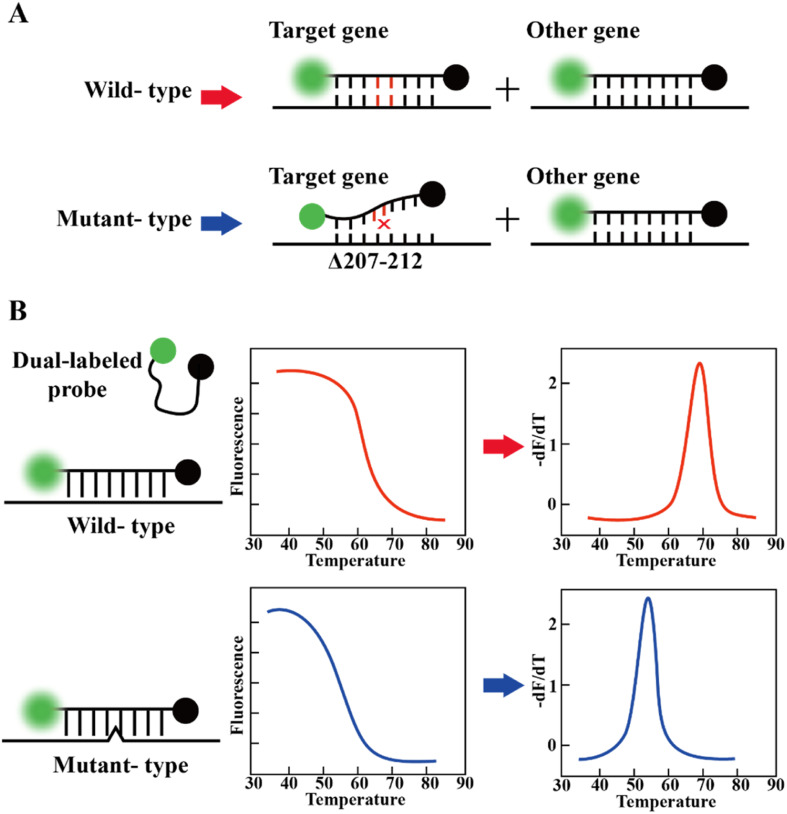
Detection methods for gene deletion and mutation in SARS-CoV-2. (A) When a deletion occurs in the S gene, the fluorescence probe cannot bind to the target gene, resulting in a negative detection for the target gene, while a positive detection for other SARS-CoV-2 genes can indicate the deletion of the target gene. (B) When a mutation occurs in the SARS-CoV-2 gene, the amplification of the mutation site produces a different amplification curve and melting curve compared to the wild-type gene.

The PCR-based diagnostic method is an effective alternative to WGS and NGS, with a flexible testing strategy and the ability for detection needs. This method can be used to assess the spread of different SARS-CoV-2 variants in the community and for genetic characterization to monitor virus evolution, while providing information for outbreak analysis.^482,483^ Several countries/area have released their RT-PCR protocols. A compilation of primer sequences was developed in China, Hong Kong, Germany, France, Thailand and the United States.^484^ The average mismatches and significant mismatches obtained from PrimerScan comparing the primers from each region with all SARS-COV-2 whole genome sequences were published on GISAID^143^ (Table 10). Since the S protein is the main target of SARS-CoV-2 nAbs, it will be more meaningful to develop RT-qPCR assays for some key mutations in the S gene, such as E484K, N501Y and 69-70del. Many manufacturers have developed kits for different sites of the S gene, such as ID SARS-CoV-2/UK/SA Variant Triplex®, PKampVariantDetect SARS-CoV-2 RT-PCR combination 1 and 3® PerkinElmer, and cobas® SARS-CoV-2 Variant Set 1 Test.^144,146^ Many recent studies have evaluated different screening kits using NGS methods, demonstrating a high level of agreement between methods.^485–487^ In addition, many scientific papers report RT-qPCR methods for S mutation detection with promising results. The detection of S-gene mutations mainly involves two strategies, including a diagnostic screening approach that utilizes negative or weakly positive S gene results (caused by deletions at nt 207–212) from multiplex RT-PCR analysis, together with positive results for other targets, to identify specific variants with deletions at this site (Fig. 16A).^488,489^ This method was extensively used when B.1.1.7 variant was prevalent in the UK and was also used for diagnosing this variant after the appearance of the B.1.1.529 variant.^486^ It is important to note that S target failure is not restricted to these two variants, given that reports indicate that it occurred in 1–5% of samples sequenced before the advent of B.1.17 VOC.^426^ Besides, target failure can also detect ORF1aΔ3675-3677 in variants (B.1.1.7, B.1.351, P.1, and C.37). Vogels *et al.* designed and validated an open-source PCR assay to detect B.1.1.7, B.1.351, and P.1 using ORF1aΔ3675-3677 as the primary target and using spikes Δ69-70 for differentiation.^490^ In addition, given that ORF1aΔ3674-3676 is present in B.1.1.529, this combined target failure strategy also has potential to be developed to differentiate between B.1.1.7 and B.1.1.529. This target failure strategy works well when the prevalence of VOC in the environment is already high, but in low prevalence settings, it is preferable to confirm the presence of both deletions by sequencing, which will be necessary to increase the confidence in the results. An alternative approach is to use S gene SNP-based analysis, which involves the design of allelic primers or specific primer probes incorporating lock nucleic acids (LNA) to target the mutation site (Fig. 16B).^491^ For example, the RT-qPCR analysis for the N501Y, 69-70del, K417N and E484K mutations developed by Vega-Magaña *et al.* and the one-step multiplex allele-specific RT-qPCR assay developed by Lee *et al.* facilitated the detection of all common and uncommon VOCs/VOIs containing L452R, E484K or N501Y mutations.^492,493^ One advantage of these methods is that they allow the rapid (<1 h) estimation of the prevalence of specific mutation-positive variants in the community, and considering that SNPs at certain sites are not only present in VOCs merely in VOIs, we should also sequence a portion of the samples for validation at the time of detection. Moreover, some RT-PCR platforms allow melting curve analysis, and based on this many genotyping methods have been developed to identify specific amino acids substitutions, such as HV69-70del, K417N, N439K, Y453F, E484K, N501Y, A570D, D614G, P681H or V1176F.^494^

**Table 10:** Summary of publicly available RT-PCR primers/probes for the analysis of SARS-CoV-2 mismatches

Region	Primer/Probe	Target gene	Sequence (5′-3′)	Primer length	Genomic region^a^	Average mismatches^b^	Significant mismatches^c^
Charité Germany	E-Sarbeco-F	E gene	ACAGGTACGTTA ATAGTTAATAGC GT	26	26 269–26 294	0.0031	—
Charité Germany	E-Sarbeco-R	E gene	ATATTGCAGCAG TACGCACACA	22	26 360–26 381		
Charité Germany	E-Sarbeco-P	E gene	ACACTAGCCATC CTTACTGCGCTT CG	26	26 332–26 357		
Charité Germany	N-Sarbeco-F	N gene	CACATTGGCACC CGCAATC	19	28 706–28 724	0.0411	0.0125
Charité Germany	N -Sarbeco-R	N gene	GAGGAACGA GAA GAGGCT TG	20	28 814–28 833		
Charité Germany	N-Sarbeco-P	N gene	ACTTCCTCAAGG AACAACATTGCC A	25	28 753–28 777		
China CDC	China-CDC-N-F	N gene	GGG GAA CTT CTC CTG CTA GAA T	22	28 881–28 902	1.1619	0.0081
China CDC	China-CDC-N-R	N gene	CAGACA TTT TGC TCTCAA GCTG	22	28 958–28 979		
China CDC	China-CDC-N-P	N gene	TTGCTGCTGCTT GACAGATT	20	28 934–28 953		
China CDC	China-CDC-ORF1-F	ORF1ab	CCCTGTGGG TTT TACACTTAA	21	13 342–13 362	0.0069	0.0030
China CDC	China-CDC-ORF1-R	ORF1ab	ACGATT GTGCAT CAGCTGA	19	13 442–13 460		
China CDC	China-CDC-ORF1-P	ORF1ab	CCGTCT GCGGTA TGTGGA AAGGTT ATGG	28	13 377–13 404		
Hong Kong University	HKU-NF	N gene	TAATCAGACAAG GAACTG ATTA	22	29 145–29 166	0.0225	0.0117
Hong Kong University	HKU-NR	N gene	CGAAGGTGT GACTTCCATG	19	29 236–29 254		
Hong Kong University	HKU-NP	N gene	GCAAATTGTGCA ATTTGCGG	20	29 177–29 196		
Hong Kong University	HKU-ORF1b-nsp141F	ORF1b	TGGGGYTTT ACRGGTAACCT	20	18 778–18 797	0.0097	0.0037
Hong Kong University	HKU-ORF1b-nsp141R	ORF1b	AACRCGCTT AACAAAGCA CTC	21	18 889–18 909		
Hong Kong University	HKU-ORF1b-nsp141P	ORF1b	TAGTTGTGATGC WATCATGACTAG	24	18 849–18 872		
Pasteur	NCoV-IP2-12669Fw	RdRp IP2	ATGAGCTTAGTC CTGTTG	18	12 690–12 707	0.0129	0.0056
Pasteur	NCoV-IP2-12759Rv	RdRp IP2	CTCCCTTTGTTG TGTTGT	18	12 780–12 797		
Pasteur	nCoV_IP2-12696 a crucial probe	RdRp IP2	AGATGTCTTGTG CTGCCGGTA	21	12 719–12 737		
Pasteur	NCoV-IP4-14059Fw	RdRp IP4	GGTAACTGGTAT GATTTCG	19	14 080–14 098	0.0131	0.0085
Pasteur	NCoV-IP4-14146Rv	RdRp IP4	CTGGTCAAG GTT AATATAGG	20	14 167–14 186		
Pasteur	NCoV-IP4-14084 probe	RdRp IP4	TCATACAAACCA CGCCAGG	19	14 105–14 123		
Public Health Thailand	WH-NIC-N-F	N gene	CGTTTGGTGGAC CCTCAGAT	20	28 320–28 339	0.0214	0.0090
Public Health Thailand	WH-NIC-N-R	N gene	CCCCACTGCGTT CTCCATT	19	28 358–28 376		
Public Health Thailand	WH-NIC-N-P	N gene	CAACTGGCAGTA ACCA	16	28 341–38 356		
US CDC	2019-nCoV-N1-F	N gene	GACCCCAAAATC AGCGAAAT	20	28 287–28 306	0.0345	0.0214
US CDC	2019-nCoV-N1-R	N gene	TCTGGTTACTGC CAGTTGAATCTG	24	28 335–28 358		
US CDC	2019-nCoV-N1-P	N gene	ACCCCGCATTAC GTTTGGTGGACC	24	28 309–28 332		
US CDC	2019-nCoV-N2-F	N gene	TTACAAACATTG GCCGCAAA	20	29 164–29 183	0.0383	0.0149
US CDC	2019-nCoV-N2-R	N gene	GCGCGACATTCC GAAGAA	18	29 213–29 230		
US CDC	2019-nCoV-N2-P	N gene	ACAATTTGCCCC CAGCGCTTCAG	23	29 188–29 210		
US CDC	2019-nCoV-N3-F	N gene	GGGAGCCTT GAATACACCAAA A	22	28 681–28 702	0.0200	0.0113
US CDC	2019-nCoV-N3-R	N gene	TGTAGCACGATT GCAGCATTG	21	28 732–28 752		
US CDC	2019-nCoV-N3-P	N gene	AYCACATTGGCA CCCGCAATCCTG	24	28 704–28 727		

a Genomic Region, Site numbering using access MN908947.3 as the reference.

b Average mismatches per sequence per assay. This value represents the overall similarity between the primers/probes and the target sequences in the database. (The data was obtained by blasting primers and probes with the database in the GISAID).

c Significant mismatches. Significant mismatches are those in the probe or the last 5 bases of a primer. These mismatches can significantly affect the specificity and sensitivity of the detection. (The data was obtained by blasting primers and probes with the database in the GISAID.)

With the increasing number of sequences becoming publicly available, many other molecular diagnostic techniques have been developed for variant detection. RT-LAMP for variant detection can also be performed directly by visual color change,^128^ fluorescence,^495^ sequence-specific probes such as Detection of Amplification by Releasing of Quenching (DARQ) and molecular beacons,^496^ or coupled to secondary molecular analysis platforms such as CRISPR and NGS (*e.g.*, LamPORE and LAMP-Seq).^497^ Currently the available protocols cannot discriminate between specific VOCs/VOIs; however, RT-LAMP technology offers certain advantages such as faster test results and requirement of less resources, while maintaining high sensitivity and specificity. CRISPR-based variant detection techniques focus on designing crRNAs with mismatches for mutation positions in known sequences^498,499^ or developing chimeric crRNAs to distinguish mutations in different positions specifically.^500^ Most of these technologies use fluorescence^501^ or LFAs^502^ for the readout of the detection results, and some have integrated technology into micro devices to develop POCT devices that integrate extraction, purification, and concentration of viral RNA, amplification and detection.^503^ CRISPR technology has the merits of easy-to-use and easy-to-set-up, which can be rapidly deployed in response to outbreaks. By combination with RT-PCR, these techniques rely on oligonucleotide primers and mutations in the target region, which may have an impact on amplification. Therefore, appropriate clinical validation studies of the protocols are also needed to assess their potential role in different settings.

## 7. Conclusions and prospect

The global epidemic is still spreading, and the SARS-CoV-2 strains are mutating. Recently, a study by Harvard University revealed that 94% of Americans were infected with COVID-19 at least once, and 97.8% had immunity to SARS-CoV-2.^504^ Although protectiveness in preventing COVID-19 has increased from 22% to 63% among the U.S. population, the rate of reinfection is increasing because the Omicron variant is extremely contagious and a large portion of Americans has stopped wearing masks. Additionally, this study also warned that as the virus evolves and the immune antibodies continuously decline, new waves of COVID-19 infections need to be cautioned. A timely and accurate identification of patients infected with SARS-CoV-2 is still the cornerstone of public health containment and mitigation of the pandemic. Presently, significant improvements have been achieved in techniques for detecting SARS-CoV-2, such as molecular assay for viral RNA and immunoassay for viral antigens or virus-specific antibodies produced by the immune system. Among them, RT-PCR is the gold standard for COVID-19 diagnosis. Mini RT-PCR devices that combine RT-PCR with different detection modalities have been deployed to address the current low sensitivity of rapid POC assays, and simultaneously analyze samples in a high-throughput format beyond the centralized laboratory.^505^ RT-dPCR has become an efficient high-throughput detection system.^506^ CRISPR/Cas-based systems have further overcome the problem of pre-test nucleic acid extraction and amplification.^507^ Moreover, diverse isothermal amplification technologies (RT-LAMP, RT-RPA, TMA) with robust detection capabilities have been implemented for rapid detection laboratories and POC applications, screening large patient populations, or rapid deployment to assess target regions^508^ Sequencing-based assays have enabled the monitoring of SARS-CoV-2 genomic sequences, especially for variant-associated detection.^509^ However, we must acknowledge that all molecular diagnostic methods are subject to a constant limitation, which is the sequence mismatch between the detection probe and the target region due to the mutations in SARS-CoV-2. Currently, false negatives caused by mutants are minimized by detecting multiple genes simultaneously or by increasing the binding to the mutant target using abbreviated primers and probes.^510,511^ During the pandemic, SARS-CoV-2 detection mainly relied on molecular diagnostic methods, especially RT-PCR, which brought many challenges globally. For example, laboratories need to procure large quantities of supplies quickly, acquire instruments, and train additional personnel. For testing, they are also faced with many challenges such as the shortage of human resources for sample collection and testing, and the unsustainable and rapid supply of swab collection resources and testing reagents. Sampling often results in crowd gathering, which poses the risk of causing virus transmission.

Antigen-based assays play an invaluable role in the rapid identification of highly infectious cases, which can generally provide rapid results without the need for complex instrumentation. However, antigen-based assays exhibit low sensitivity compared to molecular diagnostics (mean sensitivity: 56.2%, 95% CI (29.5–79.8%) for rapid antigen-based assays *vs.* 95.2%, 95% CI (86.7–98.3%) for molecular diagnostics).^512^ A possible strategy to mitigate the relatively low sensitivity of antigen-based assays is to increase the frequency of assays in the patient population over time, and to increase the chance of identifying individuals at high viral shedding stages. Serological tests are valuable for seroepidemiological studies, which contribute to ongoing outbreak investigations and the detection of suspected cases where molecular diagnostics are consistently negative or molecular diagnostics are not available. However, the performance of serological assays varies with techniques, time of onset and comparative methods. Recent meta-analyses and systematic evaluations show high levels of heterogeneity in these methods, with risks of bias and applicability.^513^ The key area of research in serology is the relevance, magnitude, and duration of protective antibody responses, especially in the case of widespread vaccination. Longitudinal studies are needed for the protective relevance of nAbs, although various neutralization assays can assess and quantify SARS-CoV-2 nAbs in serum or plasma.

Overall, RNA-, antigen-, or antibody-based assays have their respective place in diagnosing SARS-CoV-2, but ongoing research is critical to further improve them for practical applications. Eventually, the understanding of the strengths and limitations of the available methods and the development of new methods will help us unravel the unknowns of disease pathogenesis, epidemiology and transmissibility, which can restrain the SARS-CoV-2 virus.

## Data availability

Data sharing is not applicable to this article as no datasets were generated or analysed during the current study.

## Author contributions

Aihua Liu: conceptualization, funding acquisition, project administration, resources, visualization, writing – review & editing; Tao Dong: conceptualization, visualization, writing – original draft, writing – review & editing; Mingyang Wang: visualization, writing – review & editing; Junchong Liu: visualization, writing – review & editing; Pengxin Ma: visualization, writing – review & editing; Shuang Pang: visualization, writing – review & editing. Wanjian Liu: visualization, writing – review & editing.

## Conflicts of interest

There are no conflicts to declare.

## Supplementary Material
